# Serotonergic projections to the orbitofrontal and medial prefrontal cortices differentially modulate waiting for future rewards

**DOI:** 10.1126/sciadv.abc7246

**Published:** 2020-11-27

**Authors:** Katsuhiko Miyazaki, Kayoko W. Miyazaki, Gaston Sivori, Akihiro Yamanaka, Kenji F. Tanaka, Kenji Doya

**Affiliations:** 1 Neural Computation Unit, Okinawa Institute of Science and Technology Graduate University, Okinawa 904-0495, Japan.; 2 Department of Neuroscience II, Research Institute of Environmental Medicine, Nagoya University, Nagoya 464-8601, Japan.; 3 Department of Neuropsychiatry, School of Medicine, Keio University, Tokyo 160-8582, Japan.

## Abstract

Optogenetic activation of serotonergic neurons in the dorsal raphe nucleus (DRN) enhances patience when waiting for future rewards, and this effect is maximized by both high probability and high timing uncertainty of reward. Here, we explored which serotonin projection areas contribute to these effects using optogenetic axon terminal stimulation. We found that serotonin stimulation in the orbitofrontal cortex (OFC) is nearly as effective as that in the DRN for promoting waiting, while in the nucleus accumbens, it does not promote waiting. We also found that serotonin stimulation in the medial prefrontal cortex (mPFC) promotes waiting only when the timing of future rewards is uncertain. Our Bayesian decision model of waiting assumed that the OFC and mPFC calculate the posterior probability of reward delivery separately. These results suggest that serotonin in the mPFC affects evaluation of time committed, while serotonin in the OFC is responsible for overall valuation of delayed rewards.

## Teaser

Serotonergic projections to the orbitofrontal and medial prefrontal cortices modulate waiting for future rewards differently.

## INTRODUCTION

Waiting appropriately is often critical in dynamic environments to obtain future rewards. A series of studies have revealed that there is a causal relationship between activation of dorsal raphe serotonergic neurons and patience when waiting for future rewards (*1*–*7*). We further found that serotonin promotes waiting most effectively when the probability of reward delivery is high, but timing of delivery is uncertain (*8*). These results suggest that a high expectation or confidence in future rewards is necessary in order for serotonergic neural activation to promote waiting, and that the interaction of increased serotonin release and the cognitive state of the subject are crucial.

In the present study, we ask where and how serotonergic projections promote waiting for future rewards. A recent study showed that inactivation of the orbitofrontal cortex (OFC) disrupts confidence-based waiting without affecting decision accuracy (*9*). Previous recording studies have also revealed that OFC neurons encode predictions of reward outcomes (*10*, *11*). Optogenetic activation of dorsal raphe serotoninergic neurons modulates reward anticipatory responses of OFC neurons (*12*). These results suggest that the OFC may have a causal role in promoting waiting, effected by serotonin neural activation (*13*).

The nucleus accumbens (NAc) and the medial prefrontal cortex (mPFC) are also candidates as serotonergic projection targets that promote waiting. Evidence from lesion studies suggests that the core region of the NAc contributes to premature responses in the five-choice serial reaction time task (5-CSRTT) (*14*, *15*). In an intertemporal choice task, optogenetic inhibition of dorsal raphe serotonergic neurons at the decision point promoted impulsive choice, whereas optogenetic activation had the opposite effect (*16*). Excitotoxic lesions of the infralimbic PFC, the ventral part of the mPFC, induce premature responses in the 5-CSRTT (*17*). Ramping single-unit activity in the mPFC and NAc has been reported during waiting for a conditioned stimulus light in the 5-CSRTT (*18*). In the NAc and mPFC, a sustained increase in activity has been observed during waiting for delayed rewards (*18*–*21*).

In the current study, we focused on three dorsal raphe nucleus (DRN) serotonin projection target areas (the OFC, mPFC, and NAc) and optogenetically stimulated serotonergic axon terminals in these areas during waiting task performance (*22*). We tested which areas promote waiting for rewards under different levels of reward timing uncertainty (*8*). We find that serotonin stimulation in the OFC is most effective at promoting waiting and that serotonin stimulation in the NAc does not promote waiting. We also find that serotonergic stimulation in the mPFC promotes waiting only when timing of future rewards is highly uncertain. We extend our Bayesian decision model of waiting, which assumes that serotonergic neuron activation increases the prior reward probability (*8*), to reproduce the present results. The model suggests that the OFC and mPFC calculate posterior reward probability separately, using different reward timing models.

## RESULTS

### Optogenetic terminal stimulation effectively and selectively induces local serotonin efflux

To confirm the effectiveness of terminal photostimulation in increasing local serotonin efflux, we performed in vivo microdialysis experiments with an optic fiber and a microdialysis probe implanted in the OFC (fig. S1A), mPFC (fig. S1B), and NAc (fig. S1C) of Tph2-ChR2(C128S) transgenic mice (*23*, *24*).

Blue light stimulation significantly increased serotonin efflux in the OFC [*F*_8,40_ = 14.00, *P* = 1.81 × 10^−9^, one-way repeated-measures analysis of variance (ANOVA); *P* = 0.033 for time 0 versus time 5, post hoc Bonferroni correction]. Yellow light stimulation did not significantly increase serotonin efflux in the OFC (*F*_8,40_ = 1.95, *P* = 0.079, one-way repeated-measures ANOVA) (fig. S1A).

Blue light stimulation significantly increased serotonin level in the mPFC (*F*_8,40_ = 8.80, *P* = 7.59 × 10^−7^, one-way repeated-measures ANOVA; *P* = 0.023 for time 0 versus time 5, post hoc Bonferroni correction) (fig. S1B), while yellow light stimulation did not significantly increase serotonin level in the mPFC (*F*_8,40_ = 1.04, *P* = 0.42, one-way repeated-measures ANOVA) (fig. S1B). Blue light stimulation significantly increased serotonin efflux in the NAc (*F*_8,40_ = 12.21, *P* = 1.23 × 10^−8^, one-way repeated-measures ANOVA; *P* = 0.014 for time 0 versus time 5, post hoc Bonferroni correction) (fig. S1C), while yellow light stimulation did not significantly increase serotonin efflux in the NAc (*F*_8,40_ = 1.32, *P* = 0.26, one-way repeated-measures ANOVA) (fig. S1C).

To examine whether terminal photostimulation causes serotonin release in a different brain area, we implanted an optic fiber above the mPFC and measured serotonin level in the OFC by in vivo microdialysis experiment in three mice. Blue light and yellow light stimulations did not increase serotonin efflux in the OFC (blue light, *F*_8,40_ = 0.56, *P* = 0.80; yellow light, *F*_8,40_ = 1.03, *P* = 0.43, one-way repeated-measures ANOVA) (fig. S1D). This excludes the possibility of indirect activation of nonstimulated terminals by light leakage or backfiring of DRN serotonergic cell bodies.

### OFC serotonergic stimulation is highly effective at promoting patience

**Fig. 1:**
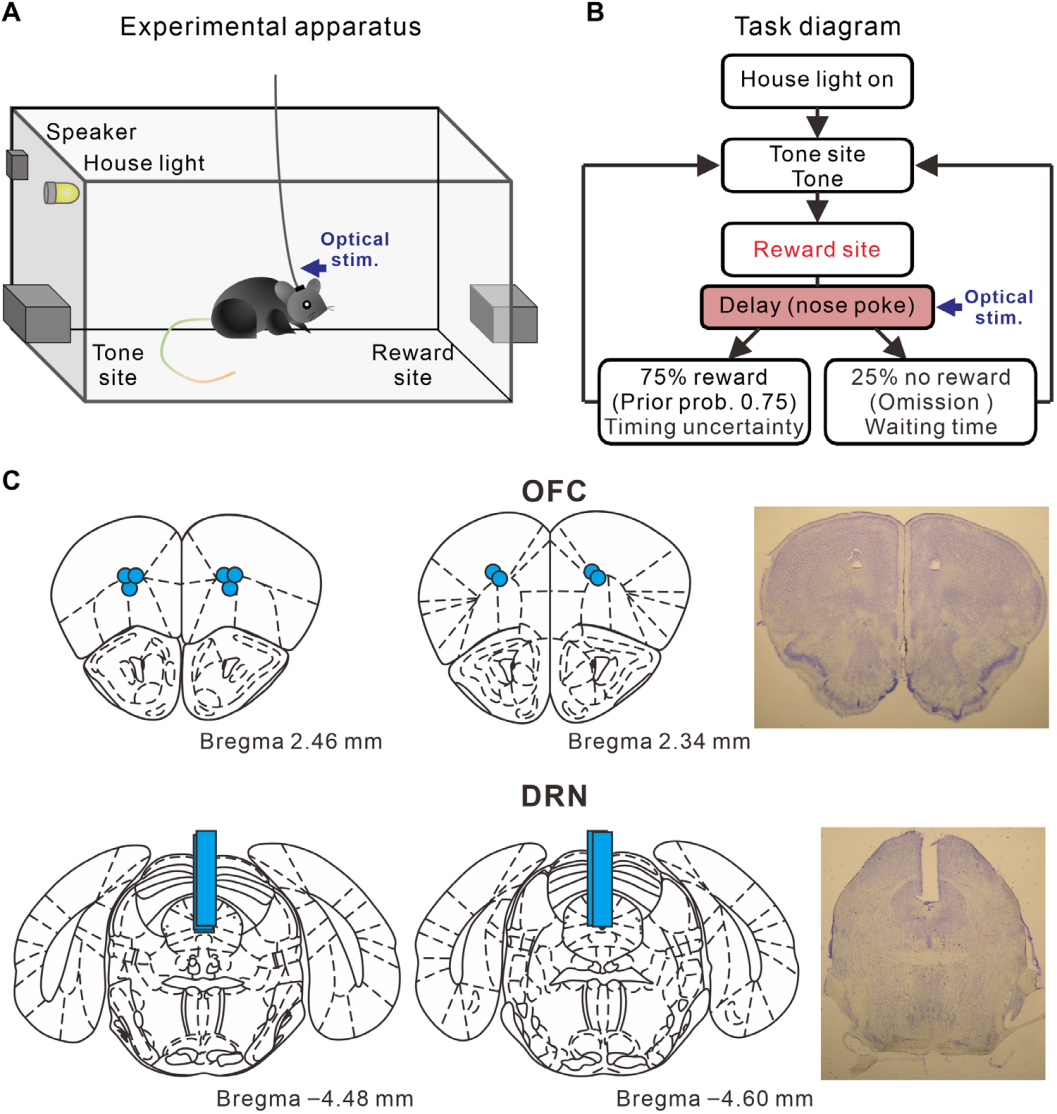
The sequential tone-food waiting task and location of optic fibers. (**A**) Experimental apparatus of the tone-food waiting task. (**B**) Diagram of the tone-food waiting task in which optogenetic stimulation was applied during waiting for delayed food, defined as a reward-delay period. Each trial started with a nose poke into tone site for 0.3 s until an 8-kHz tone was presented. After tone presentation, mice had to continue nose poking at reward site until food presentation. Seventy-five percent of trials were rewarded (i.e., prior probability of the tone-food waiting task was 0.75). Four reward-delay tests in which the timing of reward delivery was changed were introduced (i.e., change of reward timing uncertainty). To examine how serotonergic neuron activation promotes waiting for delayed rewards, we focused on waiting time in the 25% of trials with no reward (i.e., omission). Mice had to nose poke at tone site again for the next trial. (**C**) Locations of optic fibers in the OFC and DRN and representative fiber trace for the OFC and DRN. Light blue circles in the OFC represent tip positions of optic fibers. Light blue bars in the DRN represent tracks of implanted optic fibers. Coronal drawings were adapted from (*54*) with permission.

Mice [15 Tph2-ChR2(C128S) transgenic mice (*23*, *24*) and 15 wild-type mice] were trained to perform a sequential tone-food waiting task that required them to wait 0.3 s for a delayed tone (conditioned reinforcer) at a tone site and then to wait for delayed food (primary reward) at a reward site (Fig. 1, A and B). In this experiment, we prepared four reward delay conditions with a 75% reward probability: (i) fixed at 6 s (D6 test) (fig. S2A); (ii) randomly set to 4, 6, or 8 s (D4-6-8 test) (fig. S2B); (iii) randomly set to 2, 6, or 10 s (D2-6-10 test) (fig. S2C); and (iv) fixed at 10 s (D10 test) (fig. S2D). To examine how serotonergic neuron activation promotes waiting for delayed rewards, we focused on waiting time in the 25% of trials with no reward (i.e., reward omission trials).

**Fig. 2:**
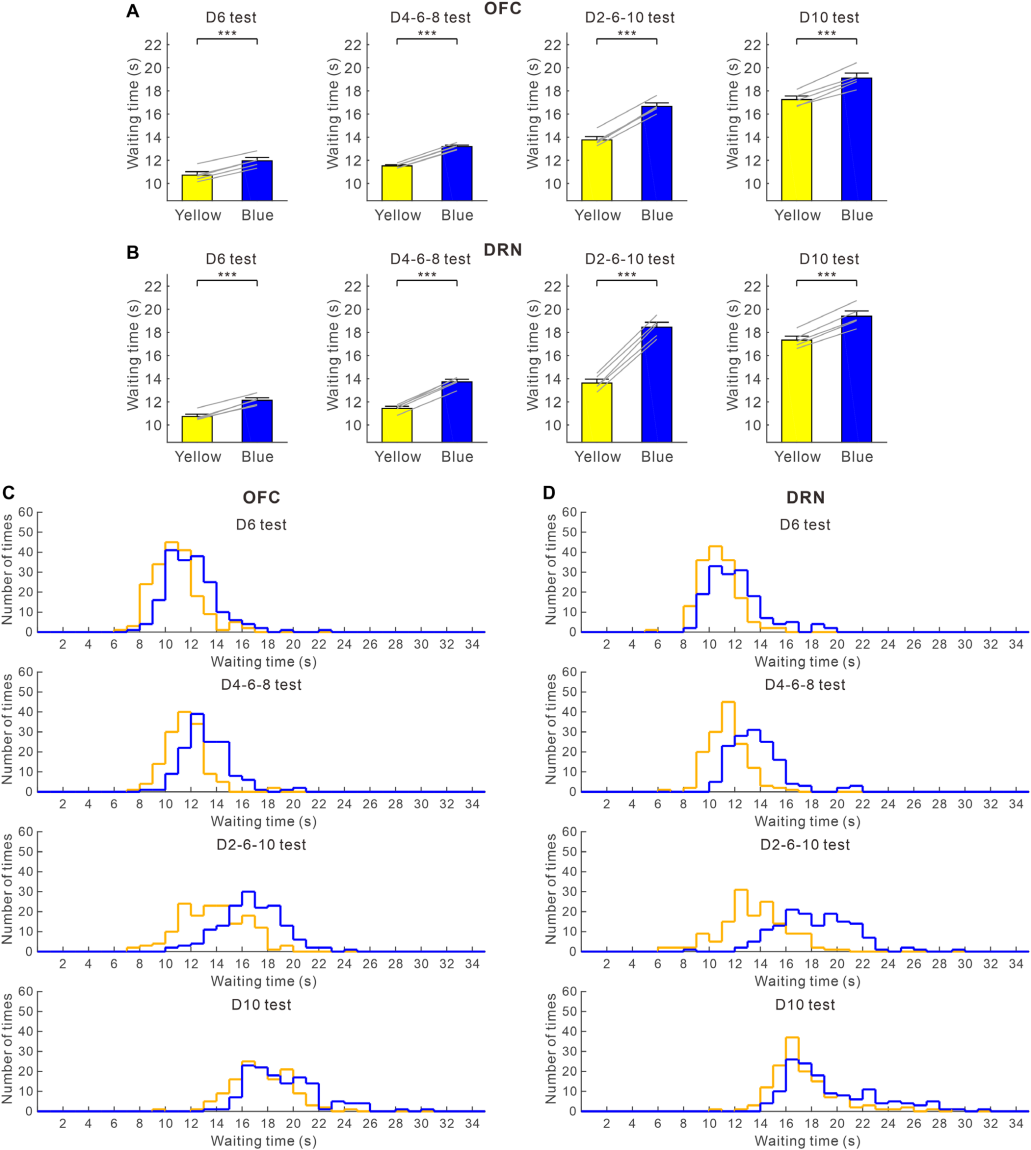
Optogenetic activation of the OFC enhances waiting in all reward delay conditions. (**A**) Average waiting time in no-activation (yellow) and activation (blue) of OFC serotonergic neuron terminals during the four reward-delay tests. Gray lines indicate waiting times for individual ChR2-expressing mice (*n* = 5). (**B**) Average waiting time in no-activation (yellow) and activation (blue) of DRN serotonergic neurons during the four reward-delay tests. Gray lines indicate waiting times for individual ChR2-expressing mice (*n* = 5). (**C**) Distribution of waiting times during omission trials in no-activation (yellow) and activation (blue) of OFC serotonergic neuron terminals during the four reward-delay tests. (**D**) Distribution of waiting times during omission trials in no-activation (yellow) and activation (blue) of DRN serotonergic neurons during the four reward-delay tests. ***P < 0.001 by paired *t* test. Error bars represent the SEM.

Five transgenic mice had optic fibers implanted into both the OFC and the DRN (Fig. 1C). In all four tests, blue light stimulation in the OFC significantly increased waiting time for omission trials, compared with waiting time in trials with yellow light stimulation (D6 test, 11.97 ± 0.28 versus 10.72 ± 0.30 s, *t*_4_ = 25.85, *P* = 1.33 × 10^−5^; D4-6-8 test, 13.18 ± 0.13 s versus 11.52 ± 0.10 s, *t*_4_ = 51.91, *P* = 8.25 × 10^−7^; D2-6-10 test, 16.67 ± 0.29 s versus 13.76 ± 0.30 s, *t*_4_ = 37.67, *P* = 2.97 × 10^−6^; D10 test, 19.11 ± 0.43 s versus 17.25 ± 0.31 s, *t*_4_ = 11.00, *P* = 3.88 × 10^−4^, paired *t* test) (Fig. 2, A and C). These effects were observed in each of the five mice tested (D6 test, *P* < 0.0037; D4-6-8 test, *P* < 0.0011; D2-6-10 test, *P* < 2.7 × 10^−5^; D10 test, *P* < 0.044, Mann-Whitney *U* test) (fig. S3, A to E). For wild-type mice with optic fibers implanted in the OFC (*n* = 5), waiting time in blue light trials did not differ significantly from that in yellow light trials, in either the D6 or D2-6-10 tests (D6 test, 11.50 ± 0.16 s versus 11.52 ± 0.22 s, *t*_4_ = 0.23, *P* = 0.83; D2-6-10 test, 14.58 ± 0.21 s versus 14.45 ± 0.11 s, *t*_4_ = 1.16, *P* = 0.31, paired *t* test) (fig. S4A). In addition, we directly compared the changes in the waiting time between transgenic mice and wild-type mice (*25*). The difference of waiting times between blue light trials and yellow light trials of transgenic mice were significantly larger than those of wild-type mice, in either D6 or D2-6-10 tests (D6 test, *t*_8_ = 10.33, *P* = 6.70 × 10^−6^; D2-6-10 test, *t*_8_ = 20.02, *P* = 4.05 × 10^−8^, unpaired *t* test) (fig. S4B).

Consistent with our previous study (*8*), in all four tests, waiting time for omission trials with serotonin activation in the DRN was significantly longer than that without serotonin activation (D6 test, 12.13 ± 0.22 s versus 10.73 ± 0.21 s, *t*_4_ = 13.72, *P* = 1.64 × 10^−4^; D4-6-8 test, 13.72 ± 0.23 s versus 11.44 ± 0.18 s, *t*_4_ = 29.58, *P* = 7.78 × 10^−6^; D2-6-10 test, 18.45 ± 0.43 s versus 13.63 ± 0.34 s, *t*_4_ = 28.45, *P* = 9.09 × 10^−6^; D10 test, 19.40 ± 0.46 s versus 17.34 ± 0.34 s, *t*_4_ = 16.77, *P* = 7.41 × 10^−5^, paired *t* test) (Fig. 2, B and D). These effects were recorded in all five mice tested (D6 test, *P* < 0.039; D4-6-8 test, *P* < 3.8 × 10^−5^; D2-6-10 test, *P* < 10^−5^; D10 test, *P* < 0.018, Mann-Whitney *U* test) (fig. S3, A to E). For wild-type mice with optic fibers implanted into the DRN (*n* = 5), waiting time in blue light trials did not differ significantly from that in yellow light trials, in either D6 or D2-6-10 tests (D6 test, 11.11 ± 0.10 s versus 11.05 ± 0.08 s, *t*_4_ = 0.71, *P* = 0.52; D2-6-10 test, 14.30 ± 0.26 s versus 14.33 ± 0.25 s, *t*_4_ = 0.38, *P* = 0.72, paired *t* test) (fig. S4C). The difference of waiting times between blue light trials and yellow light trials of transgenic mice were significantly larger than those of wild-type mice, in either D6 or D2-6-10 tests (D6 test, *t*_18_ = 12.32, *P* = 3.30 × 10^−10^; D2-6-10 test, *t*_18_ = 23.67, *P* = 5.18 × 10^−15^, unpaired *t* test) (fig. S4D).

**Fig. 3:**
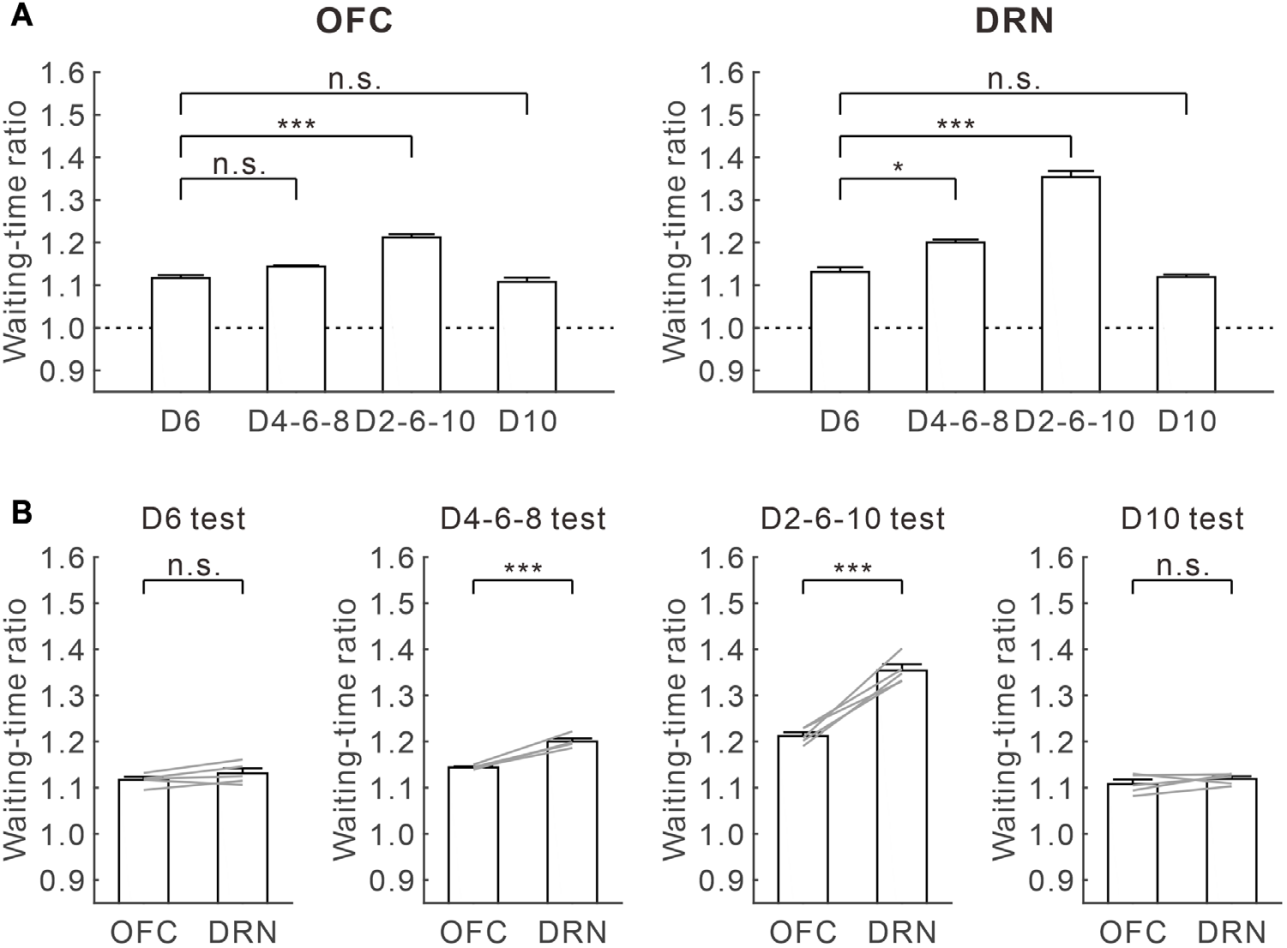
The effectiveness of OFC photostimulation in promoting patience is similar in fixed-delay tests, but less in uncertain timing compared with DRN photostimulation. (**A**) Waiting-time ratios of OFC (left) and DRN (right) optogenetic stimulation in the four reward-delay tests. *P < 0.05, **P < 0.01, and ***P < 0.001 by post hoc Bonferroni correction. n.s., not significant. Error bars present the SEM. (**B**) Comparison of waiting-time ratios between OFC and DRN optogenetic stimulation in the four reward-delay tests. Gray lines indicate waiting-time ratios for individual ChR2-expressing mice (*n* = 5). *P < 0.05, **P < 0.01, and ***P < 0.001 by paired *t* test. Error bars represent the SEM.

To quantify the effectiveness of serotonin activation in promoting waiting time during omission trials, we calculated a waiting-time ratio (waiting time with serotonin activation/waiting time without serotonin activation) for each test. Among the four delay conditions, the waiting-time ratio was largest in the D2-6-10 test in both OFC and DRN optic stimulations (for OFC, D6 test, 1.117 ± 0.007; D4-6-8 test, 1.144 ± 0.002; D2-6-10 test, 1.212 ± 0.008; D10 test, 1.108 ± 0.010, *n* = 5 mice) (*F*_3,12_ = 69.46, *P* < 10^−6^, repeated-measures ANOVA; *P* = 0.071 for D6 versus D4-6-8, *P* = 1.56 × 10^−4^ for D6 versus D2-6-10, *P* = 1.00 for D6 versus D10, post hoc Bonferroni correction) (for DRN, D6 test, 1.131 ± 0.011; D4-6-8 test, 1.200 ± 0.007; D2-6-10 test, 1.354 ± 0.014; D10 test, 1.119 ± 0.006, *n* = 5 mice) (*F*_3,12_ = 278.98, *P* < 10^−6^, repeated-measures ANOVA; *P* = 0.012 for D6 versus D4-6-8, *P* = 1.56 × 10^−4^ for D6 versus D2-6-10, *P* = 1.00 for D6 versus D10, post hoc Bonferroni correction) (Fig. 3A). The waiting-time ratio was the largest in the D2-6-10 test with optic stimulation in both the DRN and OFC in all five mice tested (for OFC, *P* < 0.065; for DRN, *P* < 0.0022, Mann-Whitney *U* test) (fig. S3, F to J).

Next, we directly compared the effectiveness of serotonin activation at promoting waiting in the OFC and the DRN. While the waiting-time ratio for DRN stimulation was significantly larger than for OFC stimulation in the D4-6-8 and D2-6-10 tests (D4-6-8 test, *t*_4_ = 11.31, *P* = 3.48 × 10^−4^; D2-6-10 test, *t*_4_ = 9.17, *P* = 7.84 × 10^−4^) (Fig. 3B), there was no significant difference in the waiting-time ratio for OFC and DRN stimulation in the D6 and D10 tests (D6 test, *t*_4_ = 1.86, *P* = 0.14; D10 test, *t*_4_ = 1.14, *P* = 0.32) (Fig. 3B). These results show that serotonin stimulation in the OFC promotes waiting in much the same fashion as DRN stimulation in fixed waiting-time tests. Serotonin stimulation in the OFC was less effective at promoting waiting than DRN stimulation, when reward timing was uncertain.

### mPFC serotonergic stimulation is effective only in high reward timing uncertainty

**Fig. 4:**
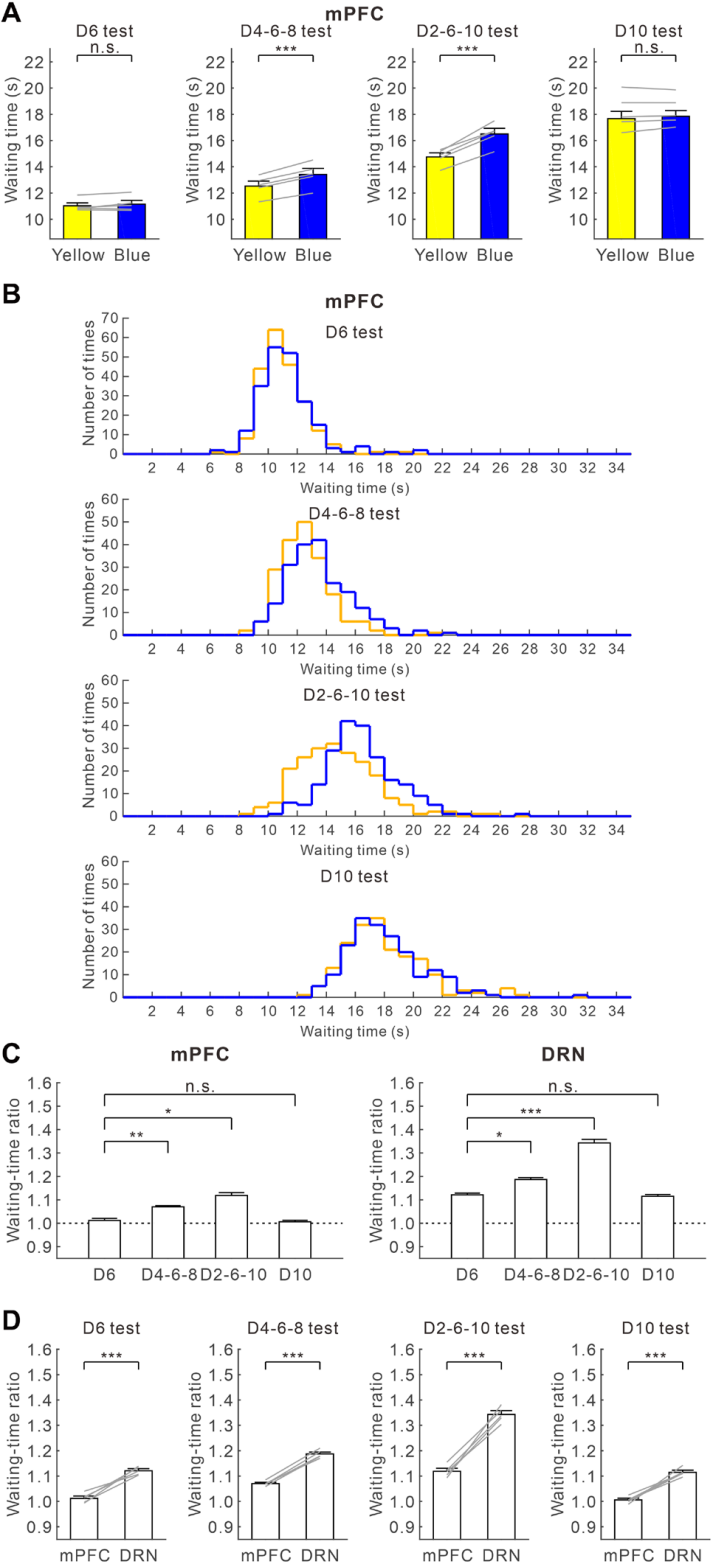
Optogenetic activation of the mPFC enhances waiting in high reward timing uncertainty. (**A**) Average waiting time in no-activation (yellow) and activation (blue) of mPFC serotonergic neuron terminals during the four reward-delay tests. Gray lines indicate waiting time for individual ChR2-expressing mice (*n* = 5). (**B**) Distribution of waiting time during omission trials in no-activation (yellow) and activation (blue) of mPFC serotonergic neuron terminals during the four reward-delay tests. ***P < 0.001 by paired *t* test. Error bars represent the SEM. (**C**) Waiting-time ratios of mPFC (left) and DRN (right) optogenetic stimulation in the four reward-delay tests. *P < 0.05, **P < 0.01, and ***P < 0.001 by post hoc Bonferroni correction. Error bars present the SEM. (**D**) Comparison of waiting-time ratios between DRN and mPFC optogenetic stimulation in the four reward-delay tests. Gray lines indicate waiting-time ratios for individual ChR2-expressing mice (*n* = 5). *P < 0.05, **P < 0.01, and ***P < 0.001 by paired *t* test. Error bars represent the SEM.

Five transgenic mice had optic fibers implanted in both the mPFC and the DRN (fig. S5A). With mPFC optogenetic stimulation, waiting time for omission trials was significantly longer than that without serotonin activation, but only in the D4-6-8 and D2-6-10 tests (D4-6-8 test, 13.42 ± 0.23 s versus 12.53 ± 0.18 s, *t*_4_ = 11.91, *P* = 2.85 × 10^−4^; D2-6-10 test, 16.50 ± 0.43 s versus 14.75 ± 0.31 s, *t*_4_ = 10.37, *P* = 4.89 × 10^−4^, paired *t* test) (Fig. 4, A and B). These effects were consistently observed in four mice during the D4-6-8 test and in all five mice tested during the D2-6-10 test (D4-6-8 test, *P* < 0.066; D2-6-10 test, *P* < 0.0073, Mann-Whitney *U* test) (fig. S5, A to E). In the D6 and D10 tests, there was no significant difference in waiting time for omission trials with and without optogenetic stimulation in the mPFC (D6 test, 11.16 ± 0.28 s versus 11.02 ± 0.23 s, *t*_4_ = 1.51, *P* = 0.21; D10 test, 18.25 ± 0.57 s versus 18.16 ± 0.68 s, *t*_4_ = 0.84, *P* = 0.45, paired *t* test) (Fig. 4, A and B). These effects were noted in each of the five mice tested (D6 test, *P* > 0.061; D10 test, *P* > 0.27, Mann-Whitney *U* test) (fig. S6, A to E).

For wild-type mice with optic fibers implanted into the mPFC (*n* = 5), waiting time in blue light trials did not differ significantly from that in yellow light trials in either the D6 or D2-6-10 tests (D6 test, 11.05 ± 0.26 s versus 11.10 ± 0.23 s, *t*_4_ = 0.69, *P* = 0.53; D2-6-10 test, 14.42 ± 0.46 s versus 14.39 ± 0.50 s, *t*_4_ = 0.20, *P* = 0.85, paired *t* test) (fig. S4E). Difference of waiting times between blue light trials and yellow light trials of transgenic mice were significantly larger than those of wild-type mice in the D2-6-10 test. There was no significant difference in the D6 test (D6 test, *t*_8_ = 1.62, *P* = 0.15; D2-6-10 test, *t*_8_ = 8.32, *P* = 3.28 × 10^−5^, unpaired *t* test) (fig. S4F).

In all four tests, waiting time in omission trials with serotonin activation in the DRN was significantly longer than without serotonin activation (D6 test, 12.42 ± 0.25 s versus 11.08 ± 0.21 s, *t*_4_ = 16.37, *P* = 8.15 × 10^−5^; D4-6-8 test, 14.49 ± 0.39 s versus 12.21 ± 0.27 s, *t*_4_ = 18.88, *P* = 4.64 × 10^−5^; D2-6-10 test, 19.47 ± 0.65 s versus 14.49 ± 0.36 s, *t*_4_ = 17.77, *P* = 5.90 × 10^−5^; D10 test, 19.74 ± 0.82 s versus 17.70 ± 0.61, *t*_4_ = 10.32, *P* = 4.97 × 10^−4^, paired *t* test) (fig. S7). These results were significant in each of the five mice tested (D6 test, *P* < 0.033; D4-6-8 test, *P* < 0.002; D2-6-10 test, *P* < 10^−6^; D10 test, *P* < 0.013, Mann-Whitney *U* test) (fig. S6, A to E).

Among the four delay conditions, the waiting-time ratio was largest in the D2-6-10 test with both mPFC and DRN optic stimulation (for mPFC, D6 test, 1.012 ± 0.009; D4-6-8 test, 1.070 ± 0.005; D2-6-10 test, 1.119 ± 0.012; D10 test, 1.006 ± 0.007, *n* = 5 mice) (*F*_3,12_ = 38.51, *P* < 10^−5^, repeated-measures ANOVA; *P* = 0.0066 for D6 versus D4-6-8, *P* = 0.024 for D6 versus D2-6-10, *P* = 1.00 for D6 versus D10, post hoc Bonferroni correction) (for DRN, D6 test, 1.121 ± 0.008; D4-6-8 test, 1.210 ± 0.008; D2-6-10 test, 1.343 ± 0.015; D10 test, 1.115 ± 0.008, *n* = 5 mice) (*F*_3,12_ = 221.85, *P* < 10^−6^, repeated-measures ANOVA; *P* = 0.016 for D6 versus D4-6-8, *P* = 5.76 × 10^−4^ for D6 versus D2-6-10, *P* = 1.00 for D6 versus D10, post hoc Bonferroni correction) (Fig. 4C). The waiting-time ratio was significantly larger in the D2-6-10 test with mPFC optic stimulation for four mice and in DRN optic stimulation for five mice (for mPFC, *P* < 0.011; for DRN, *P* < 0.0047, Mann-Whitney *U* test) (fig. S6, F to J).

In all four tests, waiting-time ratios during mPFC optogenetic stimulation were significantly smaller compared with those during DRN optogenetic stimulation (D6 test, *t*_4_ = 8.39, *P* = 1.10 × 10^−3^; D4-6-8 test, *t*_4_ = 21.81, *P* = 2.62 × 10^−5^; D2-6-10 test, *t*_4_ = 12.89, *P* = 2.09 × 10^−4^; D10 test, *t*_4_ = 9.08, *P* = 8.17 × 10^−4^, paired *t* test) (Fig. 4D). These results show that serotonergic stimulation in the mPFC does not affect waiting as strongly as such stimulation in the DRN or OFC, and it promotes waiting only when timing uncertainty of future rewards is high.

### Serotonin does not promote waiting in the NAc

**Fig. 5:**
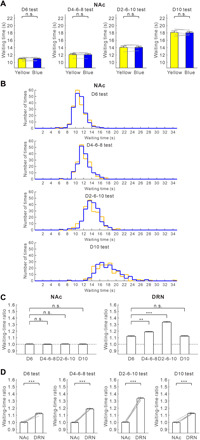
Optogenetic activation of NAc serotonergic neuron terminals does not enhance waiting. (**A**) Average waiting time during no-activation (yellow) and activation (blue) of NAc serotonin neuron terminals during the four reward-delay tests. Gray lines indicate waiting time for individual ChR2-expressing mice (*n* = 5). (**B**) Distribution of waiting times during omission trials in no-activation (yellow) and activation (blue) of NAc serotonin neuron terminals during the four reward-delay tests. ***P < 0.001 by paired *t* test. Error bars represent the SEM. (**C**) Waiting-time ratios of NAc (left) and DRN (right) optogenetic stimulation in the four reward-delay tests. *P < 0.05, **P < 0.01, and ***P < 0.001 by post hoc Bonferroni correction. Error bars present the SEM. (**D**) Comparison of waiting-time ratios of DRN and NAc optogenetic stimulation in the four reward-delay tests. Gray lines indicate waiting-time ratios for individual ChR2-expressing mice (*n* = 5). *P < 0.05, **P < 0.01, and ***P < 0.001 by paired *t* test. Error bars represent the SEM.

Optic fibers were implanted in both the NAc and the DRN in five transgenic mice (fig. S5B). In all four reward-delay tests, there was no significant difference in waiting time for omission trials with and without optogenetic stimulation in the NAc (D6 test, 10.97 ± 0.22 versus 10.97 ± 0.24 s, *t*_4_ = 0.019, *P* = 0.99; D4-6-8 test, 12.11 ± 0.32 s versus 12.13 ± 0.32 s, *t*_4_ = 0.35, *P* = 0.74; D2-6-10 test, 14.08 ± 0.33 s versus 14.07 ± 0.38 s, *t*_4_ = 0.12, *P* = 0.91; D10 test, 18.05 ± 0.35 s versus 18.11 ± 0.45 s, *t*_4_ = 0.35, *P* = 0.74, paired *t* test) (Fig. 5, A and B). This result was seen in each of the five mice tested (D6 test, *P* > 0.38; D4-6-8 test, *P* > 0.35; D2-6-10 test, *P* > 0.19; D10 test, *P* > 0.31, Mann-Whitney *U* test) (fig. S7, A to E). For wild-type mice with optic fibers implanted in the NAc (*n* = 5), waiting time in blue light trials did not differ significantly from that in yellow light trials in either the D6 or D2-6-10 tests (D6 test, 11.06 ± 0.36 s versus 11.16 ± 0.33 s, *t*_4_ = 1.44, *P* = 0.22; D2-6-10 test, 14.18 ± 0.15 s versus 14.27 ± 0.24 s, *t*_4_ = 0.66, *P* = 0.54, paired *t* test) (fig. S4G). Difference of waiting times between blue light trials and yellow light trials of transgenic mice did not differ significantly from those of wild-type mice, in either D6 or D2-6-10 tests (D6 test, *t*_8_ = 0.96, *P* = 0.37; D2-6-10 test, *t*_8_ = 0.61, *P* = 0.56, unpaired *t* test) (fig. S4H).

In all four tests, waiting time for omission trials with serotonin activation in the DRN was significantly longer than without serotonin activation (D6 test, 11.96 ± 0.31 s versus 10.67 ± 0.20 s, *t*_4_ = 11.82, *P* = 2.94 × 10^−4^; D4-6-8 test, 14.32 ± 0.53 s versus 12.06 ± 0.39 s, *t*_4_ = 16.94, *P* = 7.12 × 10^−5^; D2-6-10 test, 18.83 ± 0.28 s versus 14.11 ± 0.22 s, *t*_4_ = 32.46, *P* = 5.37 × 10^−6^; D10 test, 19.84 ± 0.34 s versus 17.67 ± 0.39 s, *t*_4_ = 5.65, *P* = 7.41 × 10^−6^, paired *t* test) (fig. S9). These results were observed in each of the five mice tested (D6 test, *P* < 0.0088; D4-6-8 test, *P* < 10^−5^; D2-6-10 test, *P* < 10^−6^; D10 test, *P* < 0.030, Mann-Whitney *U* test) (fig. S8, A to E).

Among the four delay conditions, there was no significant difference in NAc optogenetic stimulation (D6 test, 1.000 ± 0.008; D4-6-8 test, 0.998 ± 0.006; D2-6-10 test, 1.001 ± 0.007; D10 test, 0.998 ± 0.009, *n* = 5 mice) (*F*_3,12_ = 0.097, *P* = 0.96, repeated-measures ANOVA; *P* = 1.00 for D6 versus D4-6-8, *P* = 1.00 for D6 versus D2-6-10, *P* = 1.00 for D6 versus D10, post hoc Bonferroni correction), although the waiting-time ratio was largest in the D2-6-10 test in DRN optogenetic stimulation (D6 test, 1.121 ± 0.010; D4-6-8 test, 1.188 ± 0.007; D2-6-10 test, 1.335 ± 0.012; D10 test, 1.124 ± 0.007, *n* = 5 mice) (*F*_3,12_ = 146.19, *P* < 10^−6^, repeated-measures ANOVA; *P* = 0.0069 for D6 versus D4-6-8, *P* = 7.69 10^−4^ for D6 versus D2-6-10, *P* = 1.00 for D6 versus D10, post hoc Bonferroni correction) (Fig. 5C). In each of the five mice tested, NAc optic stimulation had no significant effect on waiting, although the waiting-time ratio was largest in the D2-6-10 test in DRN optic stimulation (for NAc, *P* > 0.43; for DRN, *P* < 0.015, Mann-Whitney *U* test) (fig. S8, F to J).

In all four tests, waiting-time ratios during DRN optogenetic stimulation were significantly larger compared with those during NAc optogenetic stimulation (D6 test, *t*_4_ = 8.60, *P* = 1.01 × 10^−3^; D4-6-8 test, *t*_4_ = 19.47, *P* = 4.10 × 10^−5^; D2-6-10 test, *t*_4_ = 30.39, *P* = 6.98 × 10^−6^; D10 test, *t*_4_ = 31.71, *P* = 5.89 × 10^−6^, paired *t* test) (Fig. 5D). These results show that serotonin stimulation in the NAc does not promote waiting for future rewards, in contrast to the result reported for the intertemporal choice task (*16*).

### The OFC and the mPFC may use different internal models of reward timing

To explain behavioral data regarding serotonergic terminal photostimulation theoretically, we modified the Bayesian decision model of waiting proposed to mimic effects of serotonin on waiting, depending on reward probability and timing uncertainty (*8*). The Bayesian decision model of waiting assumes that a mouse has an internal model of the timing of reward delivery and keeps estimating the probability for the trial to be rewarded while waiting. The likelihood for the trial to be rewarded declines as the mouse keeps waiting with the reward yet to come, and the posterior probability for the trial to be rewarded is estimated by multiplication with the prior probability for a rewarded trial. We propose that serotonin signals the prior probability of reward delivery.

To construct models in a data-driven way, we performed a grid search in the parameter space of the SD of the reward timing model for each test and the shift in the prior reward probability by photostimulation. The best model was selected by the fitting of the waiting time distribution measured by Kullback-Leibler divergence (see Materials and Methods).

In the first model, we assumed that shifts of prior probability by the DRN and serotonergic terminal photostimulation differ, being largest for DRN photostimulation and smaller for OFC and mPFC photostimulation. This model successfully approximates the effects of DRN and OFC photostimulation with different timing uncertainty by assuming that DRN photostimulation shifts the prior probability from 0.75 to 0.94 (fig. S10A and table S1) and that OFC photostimulation shifts the prior probability from 0.75 to 0.92 (fig. S10B and table S1). However, the effects of mPFC photostimulation are not mimicked by simply changing the prior probability. The effects of mPFC photostimulation under fixed delay conditions are too large when the prior probability shifted from 0.75 to 0.85 and the effects of uncertain timing delay conditions are too small when the prior probability is shifted from 0.75 to 0.80 (fig. S10, C and D, and table S1).

**Fig. 6:**
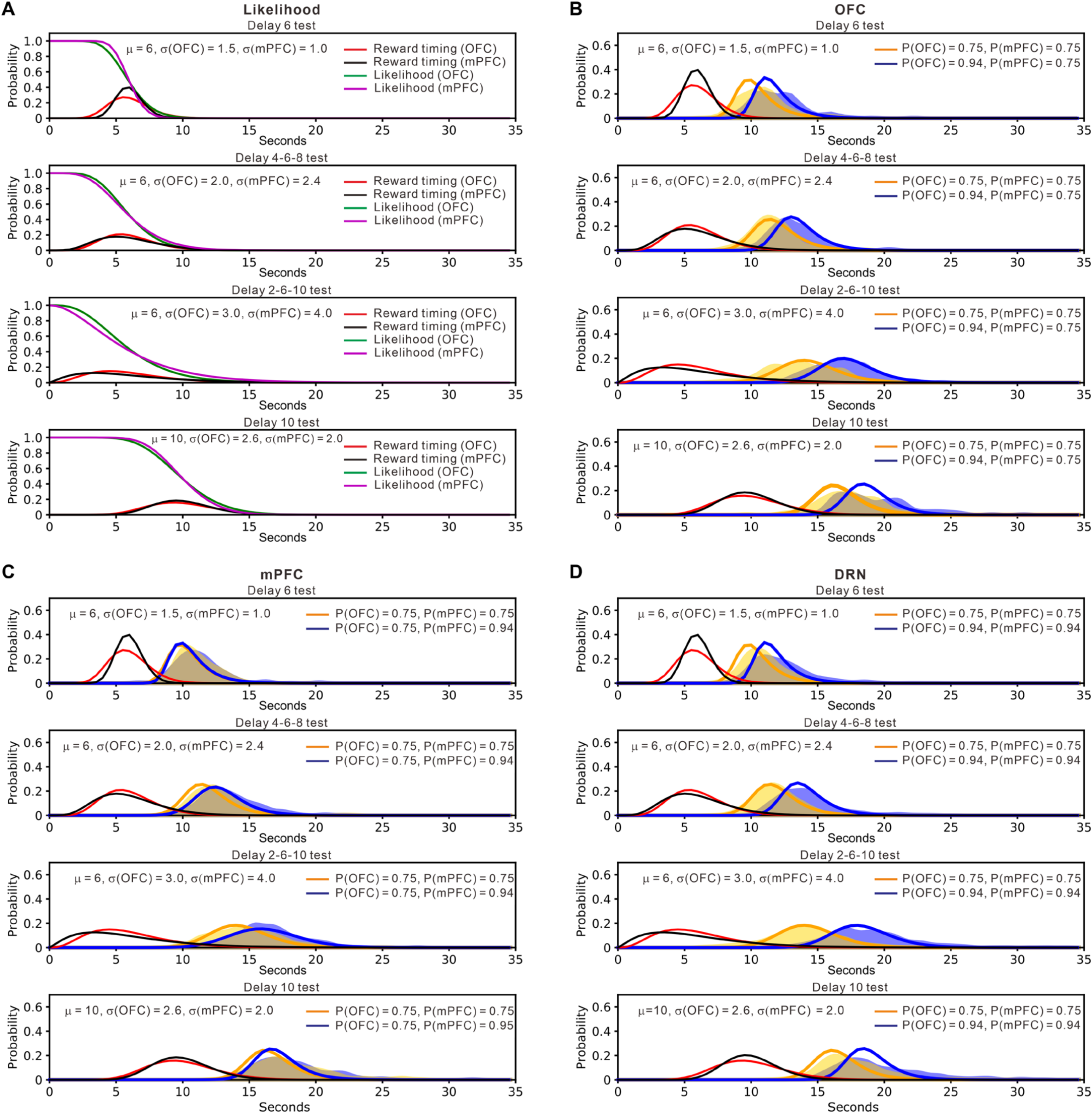
A model assuming that the OFC and mPFC independently calculate posterior probabilities reproduces features of OFC, mPFC, and DRN photostimulation. (**A**) The model assumes that the OFC and mPFC have individual probabilistic models of reward delivery timing (red lines for OFC and black lines for mPFC), which are assumed to be gamma distributions donated by μ and σ. As the time passes without reward delivery, the likelihood of a reward trial diminishes according to the cumulative density function (green lines for OFC and magenta lines for mPFC). (**B**) Simulation of waiting distribution change caused by OFC optogenetic activation. OFC photostimulation shifts the prior probability of the OFC [Prior(OFC)] from 0.75 to 0.94 and keeps the prior probability of the mPFC [Prior(mPFC)] 0.75. (**C**) Simulation of waiting distribution change by mPFC optogenetic activation. mPFC photostimulation shifts Prior(mPFC) from 0.75 to 0.94 and keeps Prior(OFC) 0.75. (**D**) Simulation of waiting distribution change by DRN optogenetic activation. DRN photostimulation shifts both Prior(OFC) and Prior(mPFC) from 0.75 to 0.94. Blue and orange lines show the time of quitting with and without increased prior probability, respectively. Yellow and blue shaded regions indicate distribution of waiting times during omission trials in no-activation and activation of serotonergic neurons, respectively.

Thus, we consider another model that assumes that the OFC and the mPFC use different internal models of reward timing to calculate posterior probabilities. The timing of reward delivery was given by a gamma distribution *G*(*t*; μ, σ^2^). While the mean (μ) was fixed to the true value (6 s in D6, D4-6-8, and D2-6-10 tests and 10 s in D10 test) the SD (σ) was set differently for the OFC (1.5, 2.0, 3.0, and 2.6 s in D6, D4-6-8, D2-6-10, and D10 tests, respectively) and for the mPFC (1.0, 2.4, 4.0, and 2.0 s, as above) (Fig. 6A). This setting implies that the timing model in mPFC is more sensitive to experienced variances in reward timing.

This model approximates the effects of OFC, mPFC, and DRN stimulation by assuming that stimulation shifts prior probabilities from 0.75 to 0.94 in the OFC, the mPFC, or both. The posterior probability for a trial to be rewarded is given by a mixture model

Posterior=α×Posterior(OFC)+(1‐α)×Posterior(mPFC)

where α is a constant that weights the contribution of OFC photostimulation.

The effect of OFC stimulation with different timing uncertainty is mimicked when OFC photostimulation shifts only the prior probability of the OFC [Prior(OFC)], but not the prior probability of the mPFC [Prior(mPFC)] and when mixing their posteriors with α = 0.8 (Fig. 6B and table S2). The effect of mPFC photostimulation is mimicked when mPFC stimulation shifts only Prior(mPFC), but not Prior(OFC) and when mixing their posteriors with α = 0.8 (Fig. 6C and table S2). The effect of DRN photostimulation is reproduced by shifting prior probabilities of both the OFC and mPFC and by mixing their posteriors with α = 0.8 (Fig. 6D and table S2). These results suggest that in the mPFC, serotonin affects evaluation of time committed, while serotonin in OFC is responsible for overall evaluation of delayed rewards.

## DISCUSSION

Our previous research revealed a causal relationship between dorsal raphe serotonergic neuron activation and patience while waiting for future rewards (*1*–*4*, *8*). In this study, using optogenetic stimulation of serotonergic terminals, we examined which DRN serotonergic projection target areas promote waiting. We found that serotonergic activation promotes waiting for delayed rewards most effectively in the OFC. OFC stimulation is as effective as DRN photostimulation when reward timing is invariant, but the effect is weaker than with DRN photostimulation when reward timing is uncertain (Fig. 3). We also found that mPFC photostimulation enhances waiting, but only when the timing of obtaining rewards is uncertain (Fig. 4). Serotonergic activation in the NAc did not enhance waiting significantly in any of the four reward delay conditions (Fig. 5). Serotonergic activation in the OFC, mPFC, and NAc contributes differently to waiting for future rewards.

### Impulsive choice and impulsive action

Numerous studies have shown that reduced levels of serotonin [5-hydroxytryptamine (5-HT)] in the central nervous system promote impulsive behaviors (*26*–*30*), including impulsive action (i.e., the failure to suppress inappropriate actions) and impulsive choice (i.e., choosing small, immediate rewards over larger, delayed rewards). In a recent study by Xu *et al.* (*16*) using the intertemporal choice task, optogenetic inhibition of dorsal raphe serotonergic neurons at the decision point promoted impulsive choice, whereas optogenetic activation had the opposite effect. Optogenetic excitation and inhibition of opsin-expressing serotonergic axonal projections in the NAc mimicked effects of manipulating dorsal raphe serotonergic cell bodies (*16*). Results from Xu *et al.* provide strong evidence that serotonergic neurons support patience by suppressing impulsive choice and that serotonergic projections to NAc make essential contributions.

In contrast, in our study, terminal photostimulation in the NAc had no significant effect on waiting. In studies of impulsive action, the 5-CSRTT is commonly used, in which nose-poke responses to one of five apertures before presentation of the stimulus light are characterized as premature responses. We previously proposed that the 5-HT system is involved in the decrease of behaviors to obtain a reward with prediction of a future reward (*13*). Because the rodents have to withhold nose-poke responses until presentation of the stimulus light (i.e., conditioned reinforcer) while they are predicting the conditioned reinforcer in the 5-CSRTT, waiting behavior with a prediction of delayed rewards in the present study is related to impulsive action.

It was previously reported that depletion of forebrain 5-HT via intraventricular administration of the selective neurotoxin, 5,7 dihydroxytryptamine (5,7-DHT), produced significant increases in premature responses in the 5-CSRTT (*31*). Optogenetic activation of dorsal raphe serotonergic neurons decreased premature responses in a 3-CSRTT (*32*). Regarding the role of the NAc in impulsive action, intra-accumbal infusion of M100907, the 5-HT2_A_ receptor antagonist, increased premature responses in the 5-CSRTT, while infusion of the 5-HT2_C_ receptor antagonist SB242084 decreased premature responses (*33*). NAc photostimulation may activate both 5-HT2_A_ and 5-HT2_C_ receptors, thereby canceling the effect on promotion of waiting time.

Previous studies also suggest that dopamine in the NAc contributes to impulsive action, but not to impulsive choice. Regarding impulsive choice, although NAc core lesions decreased the preference for large-delayed reinforcers (*34*) and systemic amphetamine administration increased the preference for large-delayed reinforcers (*35*), infusion of 6-hydroxydopamine (6-OHDA) into the NAc did not alter the effect of amphetamine (*36*). In the contrary, as for impulsive action, NAc core lesion increased premature responses in the 5-CSRTT (*14*). NAc amphetamine injection increased premature responses (*37*), and NAc 6-OHDA injection prevented the effect of amphetamine (*37*). Recently, Pisansky *et al.* (*38*) found that in mice performing the 5-CSRTT, NAc fast-spiking interneurons (FSIs) showed sustained activity in trials ending with correct responses, but FSI activity declined over time in trials ending with premature responses. They also showed that the number of premature responses increased significantly after sustained chemogenetic inhibition or temporally delimited optogenetic inhibition of NAc FSIs, without any changes in response latencies or general locomotor activity (*38*). Because NAc FSIs receive inputs from dopamine-glutamate neurons in the ventral tegmental area (*39*), NAc FSI activity related to impulsive action may be modulated by dopamine. These results indicate that dopamine in the NAc contributes to impulsive action. The results of Xu *et al.* and our results suggest that serotonin efflux in the NAc ameliorates impulsive choice, but not impulsive action.

### Effects of serotonin on the OFC and the mPFC

How do the OFC and mPFC differ in their contributions to promoting patience during waiting? Our Bayesian decision model of waiting assumes that serotonin signals the prior probability of reward delivery and that the OFC and the mPFC use different models of reward timing to compute posterior probabilities independently. Because OFC photostimulation promotes waiting more effectively than mPFC photostimulation, we assumed that the posterior probability of the OFC contributes more than that of the mPFC. To explain behavioral data showing that mPFC photostimulation promotes waiting when the timing uncertainty of reward delivery is high, we also assumed that the internal model of reward timing distribution in the mPFC has a smaller σ in fixed delay tests and a larger σ in the D4-6-8 and D2-6-10 tests, compared with those in the OFC. Are these assumptions plausible?

In electrophysiological studies, many reports have addressed the subject of neural responses in the OFC (*10*, *11*, *40*–*45*) and mPFC (*18*, *20*, *21*, *46*–*50*) during waiting for delayed rewards. These neural responses would be candidates for serotonergic promotion of waiting. The OFC is proposed to signal information about expected outcomes and to use that information to guide behavior (*11*). Optogenetic activation of OFC neurons during the waiting period improves waiting performance and lesioning or inactivation of the OFC impaired control of waiting (*44*). Serotonergic activation during waiting would modulate OFC neurons that signal reward expectancy, which could correspond to either prior or posterior probability, or both, in the Bayesian model. Because OFC neurons signal reward expectancy, the internal model of reward timing distribution gradually becomes broad when timing uncertainty of reward delivery becomes high.

There is a series of studies suggesting a role of the OFC in decision confidence (*9*, *41*, *45*). The firing rates of many single neurons in the OFC represent the confidence of decision-making when decision difficulty is manipulated by varying the distance between the stimuli and the category bound (*41*). Inactivation of the OFC disrupts waiting-based confidence reports without affecting decision accuracy (*9*). Single OFC neurons encode statistical decision confidence irrespective of the sensory modality, olfactory, or auditory, used to make a choice (*45*). These confidence signals predict confidence-guided waiting time for delayed reward (*45*). OFC neurons that respond to different timing uncertainty would be related to confidence neurons, and confidence signals would be modulated by serotonin.

Regarding the role of the mPFC during waiting for delayed rewards, involvement of the mPFC in interval timing is well supported by several lines of evidence (*46*–*51*). Disrupting the rodent mPFC increases temporal errors during a time-estimation task (*46*). Inactivation of the mPFC impairs time interval discrimination (*51*). Ramping is the most common pattern of neural activity in the mPFC during timing tasks (*47*–*49*). A subset of mPFC neurons fire in the manner of sequentially activated time cells, firing for specific periods of time during the delay of an interval discrimination task (*49*). These sequentially activated time cells showed decreasing temporal accuracy as time passed, as measured by both the width of their firing fields and the number of cells that fired during a particular part of the interval. Because the mPFC is specialized to estimate timing interval, it is possible that the timing uncertainty, σ, is smaller than in the OFC, if timing is easy to predict, as with a fixed delay. On the other hand, when timing uncertainty increases, it is possible that σ of the mPFC responds more strongly than that of the OFC, which is less specialized for timing.

Further studies are needed to clarify how neural responses during waiting for delayed rewards in the OFC and mPFC are modulated by serotonin release. Neural recording combined with optogenetic stimulation is a promising way to solve this problem.

Our Bayesian decision model of waiting proposed that the OFC and mPFC individually calculate posterior probability using serotonin. Our model may be used to evaluate serotonin function in depression model mice. Depression model mice include both serotonin-selective reuptake inhibitor (SSRI)–responsive model mice (*52*) and SSRI nonresponsive model mice (*53*). Dysfunction of the serotonergic system would differ between these two depression model mouse lines. The Bayesian decision model of waiting may evaluate which parameters are affected by serotonergic neuron activation in the DRN and serotonin projection areas in depression model mice. These data may reveal which neural circuits are impaired in each depression model.

## MATERIALS AND METHODS

### Animals

All experimental procedures were performed in accordance with guidelines established by the Okinawa Institute of Science and Technology Experimental Animal Committee. Serotonergic neuron-specific ChR2(C128S)-expressing mice were produced by crossing Tph2-tTA mice with tetO-ChR2(C128S)-EYFP knock-in mice (*23*, *24*). Twenty-seven male bigenic and 15 wild-type C57BL/6J adult mice, aged >4 months at the beginning of the behavioral training period, were used in the study. Animals were housed with one mouse per cage at 24°C on a 12-hour:12-hour light:dark cycle (lights on 07:00 to 19:00). Fifteen bigenic mice (5 with implanted optic fibers in the OFC and DRN, 5 with implanted optic fibers in the mPFC and DRN, and 5 with implanted optic fibers in the NAc and DRN) and 15 wild-type animals were used to generate the behavioral data reported here. Twelve bigenic mice (three with one optic fiber and one microdialysis probe implanted in the OFC, three with one optic fiber and one microdialysis probe implanted in the mPFC, three with one optic fiber and one microdialysis probe implanted in the NAc, and three with one optic fiber implanted in the mPFC and one microdialysis probe implanted in the OFC) were used for microdialysis experiments. Training and test sessions were conducted during the light period, 5 days per week. Mice were deprived of food in their home cage and received their daily food ration during experimental sessions only (approximately 2 to 3 g/day). Food was freely available during weekends and was removed more than 15 hours before experimental sessions started. Water was freely available in the home cage.

### Surgery

After mice had mastered the sequential tone-food waiting task, they were anesthetized with isoflurane (1.0 to 3.0%) and fixed in a stereotaxic frame (Narishige). Optic fibers were stereotaxically implanted above the OFC, the mPFC, the NAc, and the DRN. For the OFC, two optic fibers [300 μm diameter, 0.37 numerical aperture (NA), 4 mm length, Doric Lenses] were bilaterally implanted with a 20° angle from caudal to rostral (from the bregma: posterior, +2.4 mm; lateral, ±0.95 mm; ventral, −2.0 mm). For the mPFC, one optic fiber (400 μm diameter, 0.48 NA, 4 mm length, Doric Lenses) was implanted with a 20° angle from caudal to rostral (from the bregma: posterior, +1.9 mm; lateral, 0 mm; ventral, −1.3 mm). For the NAc, two optic fibers (300 μm diameter, 0.37 NA, 4 mm length, Doric Lenses) were bilaterally implanted with a 20° angle from caudal to rostral (from the bregma: posterior, +1.3 mm; lateral, ±0.95 mm; ventral, −3.8 mm). For the DRN, one optic fiber (400 μm diameter, 0.48 NA, 4 mm length, Doric Lenses) was implanted (from the bregma: posterior, +4.5 mm; lateral, 0 mm; ventral, −2.6 mm). Optic fibers were fixed with ultraviolet (UV) adhesive (LOCTITE 4305, Henkel) and clear dental cement (Super-Bond, Sun Medical). Animals were housed individually after surgery and allowed to recover for at least 1 week.

For microdialysis experiments, both an optic fiber and a guide cannula are simultaneously implanted above the OFC, the mPFC, and the NAc. For the OFC, one optic fiber (300 μm diameter, 0.37 NA, 4 mm length, Doric Lenses) was implanted with a 20° angle from caudal to rostral (from the bregma: posterior, +2.4 mm; lateral, +0.95 mm; ventral, −2.0 mm). One guide cannula (AG-4; Eicom) was implanted above the OFC (from the bregma: posterior, +2.6 mm; lateral, +0.95 mm; ventral, −2.0 mm). For the mPFC, one optic fiber (400 μm diameter, 0.48 NA, 4 mm length, Doric Lenses) was implanted with a 20° angle from caudal to rostral (from the bregma: posterior, +1.9 mm; lateral, 0 mm; ventral, −1.3 mm). One guide cannula (AG-4; Eicom) was implanted above the mPFC (from the bregma: posterior, +2.1 mm; lateral, +0.3 mm; ventral, −1.3 mm). For the NAc, one optic fiber (300 μm diameter, 0.37 NA, 4 mm length, Doric Lenses) was implanted with a 20° angle from caudal to rostral (from the bregma: posterior, +1.3 mm; lateral, +0.95 mm; ventral, −3.8 mm). One guide cannula (AG-4; Eicom) was implanted above the NAc (from the bregma: posterior, +1.5 mm; lateral, +0.95 mm; ventral, −3.8 mm). Optic fibers and guide cannulas were fixed with UV adhesive and clear dental cement. A dummy cannula (AD-4; Eicom) was inserted into the guide cannula and secured to the guide cannula with a cap nut (AC-1; Eicom) to prevent infection and occlusions. Animals were housed individually after surgery and allowed to recover for at least 1 week.

### In vivo microdialysis and optical stimulation

Each mouse was anesthetized with isoflurane (1.0 to 3.0%). A dialysis probe (A-I-4-01; length 1 mm, outer diameter 0.22 mm, 50,000 molecular weight cutoff, Eicom) was carefully inserted into the guide cannula of the OFC, mPFC, and NAc. The probe was secured to the guide cannula with a screw. The dialysis probe was perfused at a constant flow rate of 2 μl/min with Ringer’s solution (147.2 mM NaCl, 4.0 mM KCl, and 2.2 mM CaCl_2_; Wako, Osaka, Japan). To augment levels of serotonin in the dialysate, the perfusate of the dialysis probe contained a low concentration of citalopram (1 μM) (Sigma-Aldrich) for measuring serotonin levels in the OFC and mPFC. Extracellular serotonin levels were measured by high-performance liquid chromatography using electrochemical detection every 5 min (*1*, *2*). To examine the effect of optogenetic stimulation on serotonin neurons, the following two light conditions were used: continuous yellow light and continuous blue light. In continuous yellow light condition, 10 s of yellow light stimulation was followed by 10 s of no light, and this 20-s sequence was repeated 15 times for 5 min. In the continuous blue light condition, 10 s of blue light stimulation was followed by 2 s of yellow light and 8 s of no light, and this 20-s sequence was repeated 15 times for 5 min. Blue light power intensities at the tips of optic fibers were measured with a power meter (LPM-100; BRC) and were 1 mW for the OFC and NAc and 2 mW for the mPFC. Yellow light power intensities at the tips of optic fibers were 1 mW for the OFC and NAc and 2 mW for the mPFC.

### Reconstruction of optical stimulation sites

Mice were deeply anesthetized with sodium pentobarbital (100 μg/g, intraperitoneally) and then perfused with 0.9% NaCl, followed by 10% formalin. Their brains were removed and stored in 10% formalin for a minimum of 24 hours before being sliced into 60-μm coronal sections. Cresyl violet staining was used to help verify placements of optic fiber tracks (Fig. 1C and fig. S5).

### Behavioral apparatus and training

Animal training was performed as described previously (*4*, *8*). A free operant task that we designated as a sequential tone-food waiting task was used. Mice were individually trained and tested in an operant-conditioning box (Med Associates) measuring 21.6 cm by 17.8 cm by 12.7 cm. The box could be illuminated with a single 2.8-W light located in the top center of the rear wall. One speaker was positioned in the top right side of the rear wall. Three 2.5 cm × 2.5 cm apertures were positioned 2 cm above the floor. The rear stainless steel wall of the chamber contained one aperture defined as the tone site. On the front wall, two apertures defined as food sites were positioned 7 cm apart. Both apertures on the front wall were connected to a food pellet dispenser that delivered a food pellet (20 mg) to these apertures. In all experiments, only the right food site was used, and the left aperture was covered to prevent nose poking. An infrared photobeam crossed the entrances of all apertures to detect nose pokes and was positioned 0.5 cm behind the aperture and 1 cm above the bottom of it. The operant box was illuminated by the aforementioned light and was enclosed in a sound-attenuating chamber equipped with a ventilation fan. When a mouse poked its nose through an aperture in the back or front wall, the infrared photobeam was interrupted, detecting the response. A tone-site nose poke induced an 8-kHz tone (0.5 s, 85 dB) from the speaker. At the food site, a small food pellet (20 mg) was delivered to the aperture by the food dispenser. All experimental data were recorded with an EPSON personal computer connected to the operant box via an interface using MED-PC IV software (Med Associates).

The beginning of the sequential tone-food waiting task was signaled by turning on the light, and termination was indicated by turning it off. The behavioral instrumental response in this task was for a mouse to hold its nose in either the tone-site aperture while waiting for the conditioned reinforcer tone or the reward-site aperture while waiting for a food reward. This task required mice to perform alternate visits and nose pokes between the sites. A mouse initiated a trial by nose poking so as to achieve continuous interruption of the photobeam at the tone site for a delay until the tone was presented, signaling that a food reward was available at the reward site. After the tone was presented, mice were required to continue nose poking at the reward site until the reward was delivered. The delay period that preceded the tone was called the tone delay and that which preceded the food was termed the reward delay. During the initial training period, the tone and reward delays were fixed at 0.2 s.

Two types of error could occur in this task: the tone-wait error and the reward-wait error. Tone-wait and reward-wait errors occurred when a mouse failed to keep its nose in a fixed posture while waiting for the tone or food, respectively, during delay periods. After a tone-wait error, the mouse could restart the trial until it succeeded in waiting for the tone. A trial ended when the mouse received food or a food-wait error. During a trial, a tone-wait error could occur multiple times. In contrast, a reward-wait error could only occur once. Occurrences of tone and reward-wait errors were not signaled. Mice could start the next trial at any time after food consumption or after receiving a reward-wait error. Mice were trained daily for 2 hours. Criteria for task performance were that mice could get more than 60 food pellets within 2 hours and mice could perform the task with a success rate of reward acquisition [rewards number/(rewards number + reward-wait error number) × 100%) > 90%] for three successive days. All trained mice achieved these criteria in 2 weeks or less.

### In vivo optical stimulation during the sequential tone-food waiting task

During the test session, external optic fibers (300 μm diameter, 0.37 NA, bilaterally for OFC and NAc; 400 μm diameter, 0.48 NA, unilaterally for mPFC and DRN, Doric Lenses) were coupled to implanted optic fibers using zirconia sleeves. Optic fibers were connected to an optic swivel (Doric Lenses) that allowed unrestricted in vivo illumination. The optic swivel was connected to 470-nm blue and 590-nm yellow light-emitting diodes (LEDs) (470 nm, 35 mW; 590 nm, 10 mW, Doric Lenses) to generate blue and yellow light pulses through the optic fiber (960 μm diameter, 0.48 NA, Doric Lenses). Blue light intensities at the tips of optic fibers were 1 mW for the OFC and NAc, 1.5 mW for the DRN, and 2 mW for the mPFC. Yellow light intensities at the tips of optic fibers were 1 mW for the OFC and NAc, and 2 mW for the mPFC and DRN. The LED was controlled by transistor-transistor logic (TTL) pulses generated with an MED-PC IV.

### Serotonergic terminal stimulation experiment

After recovery from surgery, mice were retrained daily for 2 hours on the sequential food-water waiting task, in which the reward delay was gradually extended up to 6 s (1-s delay for 1 day, 2-s delay for 1 day, 4-s delay for 1 day, and 6-s delay until achieving the criteria). Criteria for task performance were that mice could get more than 60 food pellets within 2 hours and could perform the task with >90% reward acquisition [rewards number/(rewards number + reward-wait error number) × 100%] for three successive days. The tone delay was fixed at 0.3 s.

To examine which serotonin projecting areas promote waiting for rewards, we used four delayed-reward tests, as described previously, which showed that the promotion of waiting by serotonin was more effective when the timing uncertainty of future rewards was high (*8*). Four reward-delay tests, in which the timing of reward delivery was changed, provided rewards with a 75% probability: (i) The reward delay was fixed at 6 s (D6 test) (fig. S2A); (ii) the reward delay was randomly set to 4, 6, or 8 s (D4-6-8 test) (fig. S2B); (iii) the reward delay was randomly set to 2, 6, or 10 s (D2-6-10 test) (fig. S2C); and (iv) the reward delay was fixed at 10 s (D10 test) (fig. S2D). Each test lasted 50 min or until a mouse completed 40 trials. The tone delay was 0.3 s. The 0.5-s tone (8 kHz) was fixed in all four reward delay conditions. Removing the nose more than 500 ms before the end of the reward delay caused a reward-wait error such that no reward was presented.

Trials in which serotonergic neurons were or were not optogenetically stimulated were called serotonin activation trials or serotonin no-activation trials, respectively (fig. S2). For serotonin activation trials, blue light was continuously applied while waiting for a reward and was terminated when mice received food or made a reward-wait error. Continuous blue light stimulation was followed by 1 s of yellow light stimulation, just after termination of the blue light (fig. S2). For serotonin no-activation trials, yellow light was continuously applied while waiting for a reward and terminated when mice received food or made a reward-wait error. Continuous yellow light stimulation was followed by 1 s of yellow light stimulation just after the termination of yellow light stimulation (fig. S2). In the D4-6-8 and D2-6-10 tests, the eight trial patterns (two light conditions × four delay lengths) were randomly selected without repetition until all items were selected, and then this selection was repeated five times. In the D6 and D10 tests, eight trials (three fixed delay with serotonin activation, one omission with serotonin activation, three fixed delay without serotonin activation, and one omission without serotonin activation) were randomly selected without repetition until all items were selected, and then this selection was repeated five times.

We executed D6, D4-6-8, D2-6-10, and D10 test sessions in this order. In each reward-delay test session, the first day was a training session followed by 4 days of recording sessions in which 2 days of one serotonin projecting area (OFC, mPFC, or NAc) photostimulation and 2 days of DRN photostimulation were randomly selected. In each one-day recording session, photostimulation was applied to only one area. Each reward-delay test session lasted 1 or 2 weeks. One-day recording sessions consisted of at least two reward-delay tests.

### Data analysis

Sample sizes were similar to those used in our previous study (*4*, *8*). To examine how serotonergic neuron activation promotes waiting for delayed rewards, we focused on waiting time during omission trials. To quantify effectiveness of serotonergic activation in promoting waiting during omission trials, we calculated the waiting-time ratio (waiting time during serotonin activation trials/waiting time during serotonin no-activation trials) for each test. For individual mice, statistically significant differences (waiting time or waiting-time ratio) between two groups were assessed using the Mann-Whitney *U* test. To compare waiting time in serotonin activation and serotonin no-activation by within animal averages, we used paired *t* tests. To compare difference of waiting times in serotonin activation and serotonin no-activation by between animal averages, we used unpaired *t* tests. Normality of data for paired *t* test, unpaired *t* tests, and one-way repeated-measures ANOVA was assessed using the Shapiro-Wilk test. One-way repeated-measures ANOVA tests followed by Bonferroni corrections for multiple comparisons were used for analysis of the waiting-time ratio for individual animal averages. To compare the waiting-time ratio in DRN photostimulation and in serotonergic projecting areas for individual animal averages, we used paired *t* tests. In a very small number of omission trials, mice removed their noses from the reward site within 2 s (in the D6 test, two for serotonin no-activation trials in OFC photostimulation, two for serotonin no-activation trials in NAc photostimulation, three for serotonin activation trials in DRN photostimulation; in the D4-6-8 test, one for serotonin a no-activation trial in OFC photostimulation, three for serotonin no-activation trials in mPFC photostimulation, two for serotonin no-activation trials and two for serotonin activation trials in NAc photostimulation, and one for a serotonin no-activation trial and three for serotonin activation trials in DRN photostimulation; in the D2-6-10 test, two for serotonin no-activation trials and three for serotonin activation trials in NAc photostimulation and two for serotonin activation trials in DRN photostimulation; in the D10 test, four for serotonin no-activation trials and two for serotonin activation trials in OFC photostimulation, two for serotonin no-activation trials and one for a serotonin activation trial in mPFC photostimulation, one for a serotonin no-activation trial and one for a serotonin activation trial in NAc photostimulation, and seven for serotonin no-activation trials and two for serotonin activation trials in DRN photostimulation). These data were excluded from subsequent analyses. Statistical analyses were performed using SPSS or MATLAB (MathWorks).

### Bayesian decision model of waiting

The Bayesian decision model of waiting proposed in this study was modified from that described previously (*8*). Each trial had a hidden state *X* = {reward, no-reward}, and for a reward trial, the timing of reward delivery was given by a gamma distribution *G*(*t*; μ, σ^2^). Given an observation that a reward had not been delivered by time *t*, the likelihood for a reward trial was 1 – *f*(*t*; μ, σ^2^), where *f* is the cumulative density function of gamma distribution, whereas the likelihood for a no-reward trial was 1. The posterior probability for a reward trial given observation of no reward by time *t* was

$$P(\text{reward}|t)=P(\text{reward})*[1-f(t;\mu ,\sigma^{2})]/[P(\text{reward})*(1-f(t;\mu ,\sigma^{2}))+P(\text{no-reward})]$$

where *P*(reward) and *P*(no-reward) are prior probabilities of reward and no-reward trials.

The expected reward to continue waiting was *V*(wait|*t*) = *P*(reward|*t*) for a unit of reward, while the expected reward for quitting was *V*(quit|*t*) = −0.05 as no reward was obtained by quitting. Assuming a softmax action selection, the choice probability to continue waiting at time *t* was

P(wait∣t)=1/(1+exp[–β*(P(reward∣t)–V(quit∣t))])

where β is the inverse temperature parameter regulating the stochasticity of choice. The distribution of the time of quitting *P*_quit_(*t*) was given by sequential decisions

$$\begin{array}{c} P_{\text{wait}}(0)=1\\ P_{\text{wait}}(t)=P_{\text{wait}}(t–\tau )*P(\text{wait}|t)\\ P_{\text{quit}}(t)=P_{\text{wait}}(t–\tau )*[1–P(\text{wait}|t)] \end{array}$$

where *P*_wait_(*t*) is the probability of continuing to wait until time *t* and τ is the interval of repeated decisions to wait or to quit. In Fig. 6, we used parameters τ = 0.5 s and β = 15. To estimate the change in the prior probability of a reward trial that serotonin activation exerts, we compared the model output to behavior data. To do this, we first performed a grid search of best-fitting parameter values that minimized the averaged Kullback-Leibler divergence measured between the simulation output and the estimated probability density function (kernel density estimation) across all light photostimulations and reward-delay tests (time bin, 0.5 s; bandwidth, 0.5). The code of the Bayesian waiting decision model was written in Python.

## Supplementary Material

http://advances.sciencemag.org/cgi/content/full/6/48/eabc7246/DC1

Adobe PDF - abc7246_SM.pdf

Serotonergic projections to the orbitofrontal and medial prefrontal cortices differentially modulate waiting for future rewards

## SUPPLEMENTARY MATERIALS

Supplementary material for this article is available at http://advances.sciencemag.org/cgi/content/full/6/48/eabc7246/DC1

## Appendix

View/request a protocol for this paper from *Bio-protocol*.
