# Supplementary material for: Serotonergic projections to the orbitofrontal and medial prefrontal cortices differentially modulate waiting for future rewards
Source: Sci Adv. 2020 Nov 27;6(48):eabc7246. doi: 10.1126/sciadv.abc7246 (PMC7695476; doi:10.1126/sciadv.abc7246)
Supplement: http://advances.sciencemag.org/cgi/content/full/6/48/eabc7246/DC1 [file supp_6_48_eabc7246__index.html]

Science Advances | Science AdvancesAAASSearchScience AdvancesMenu

## Supplementary Materials

# Serotonergic projections to the orbitofrontal and medial prefrontal cortices differentially modulate waiting for future rewards

Katsuhiko Miyazaki, Kayoko W. Miyazaki, Gaston Sivori, Akihiro Yamanaka, Kenji F. Tanaka, Kenji Doya

Download Supplement

**This PDF file includes:**

- Figs. S1 to S10
- Tables S1 and S2

**Files in this Data Supplement:**

- Adobe PDF - abc7246\_SM.pdf
