# Supplementary material for: Serotonergic projections to the orbitofrontal and medial prefrontal cortices differentially modulate waiting for future rewards
Source: Sci Adv. 2020 Nov 27;6(48):eabc7246. doi: 10.1126/sciadv.abc7246 (PMC7695476; doi:10.1126/sciadv.abc7246)
Supplement: http://advances.sciencemag.org/cgi/content/full/6/48/eabc7246/DC1 [file supp_6_48_eabc7246__1.pdf]

Science Advances
AAAS

advances.sciencemag.org/cgi/content/full/6/48/eabc7246/DC1

Supplementary Materials for

Serotonergic projections to the orbitofrontal and medial prefrontal cortices differentially modulate waiting for future rewards

Katsuhiko Miyazaki*, Kayoko W. Miyazaki, Gaston Sivori, Akihiro Yamanaka, Kenji F. Tanaka, Kenji Doya

*Corresponding author. Email: miyazaki@oist.jp

Published 27 November 2020, *Sci. Adv.* **6**, eabc7246 (2020)
DOI: 10.1126/sciadv.abc7246

This PDF file includes:

Figs. S1 to S10
Tables S1 and S2

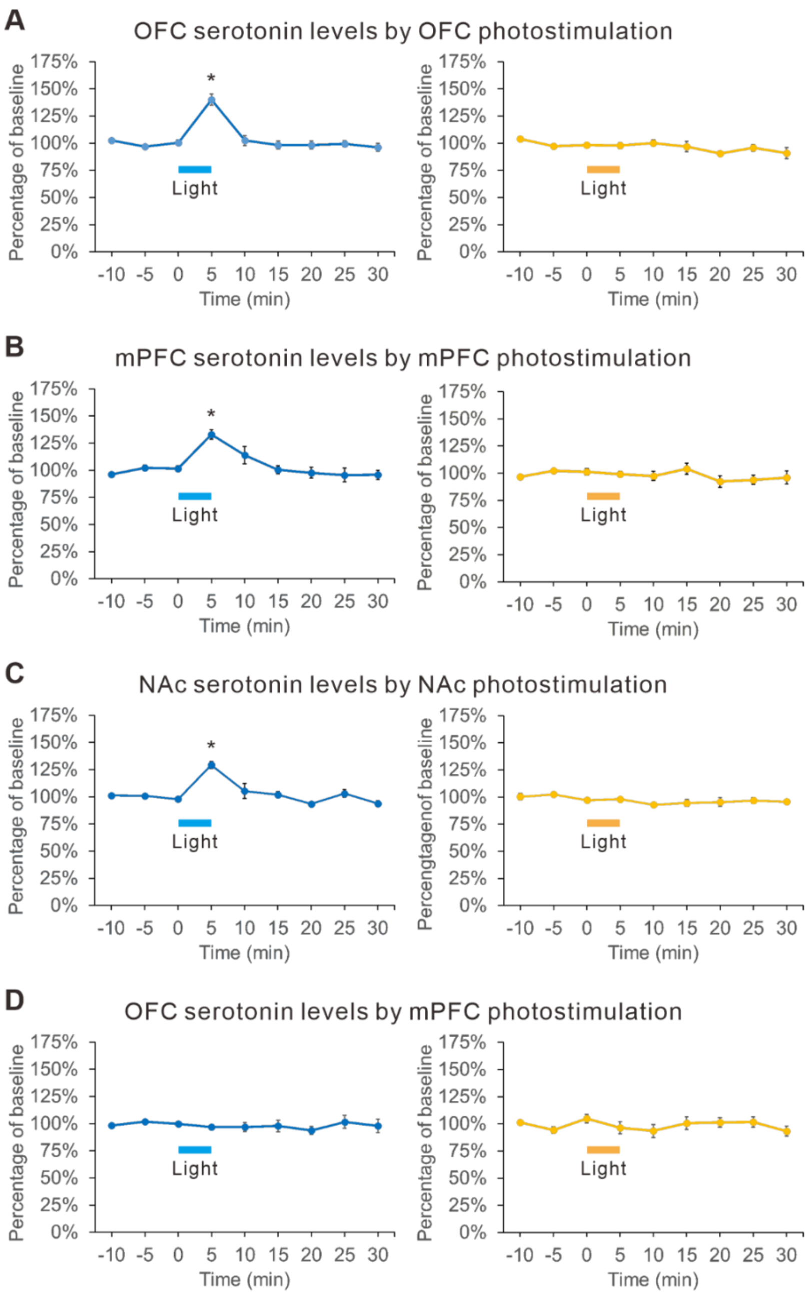

Fig. S1. Optogenetic terminal stimulation effectively and selectively induces local serotonin efflux. (**A-C**) Serotonin levels significantly increased after blue light stimulation. Yellow light stimulation did not significantly increase serotonin levels. (**A**) Time course of changes in extracellular serotonin levels in the OFC by blue light stimulation (left) and yellow light stimulation (right) in the OFC are shown (n = 6 from there transgenic mice). (**B**) Time course of changes in extracellular serotonin levels in the mPFC by blue light stimulation (left) and yellow light stimulation (right) in the mPFC are shown (n = 6 from there transgenic mice). (**C**) Time course of changes in extracellular serotonin levels in the NAc by blue light stimulation (left) and yellow light stimulation (right) in the NAc are shown (n = 6 from there transgenic mice). (**D**) Time course of changes in extracellular serotonin levels in the OFC by blue light stimulation (left) and yellow light stimulation (right) in the mPFC are shown (n = 6 from there transgenic mice). Blue and yellow light stimulation did not significantly increase serotonin levels. *P < 0.001 by post hoc Bonferroni correction. Error bars represent the s.e.m.

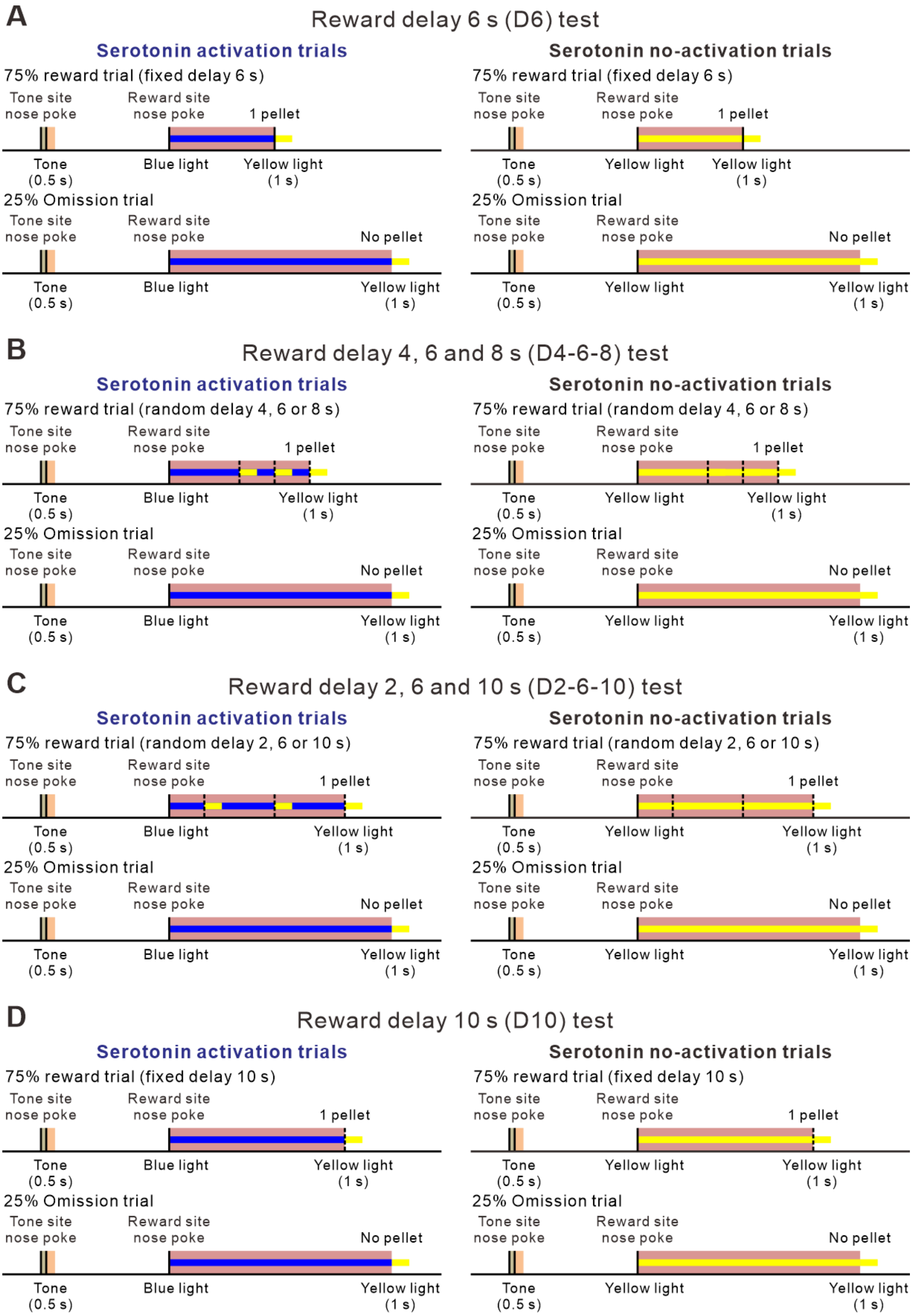
A
Reward delay 6 s (D6) test
Serotonin activation trials
75% reward trial (fixed delay 6 s)
Tone site nose poke
Reward site nose poke
1 pellet
Tone (0.5 s)
Blue light
Yellow light (1 s)
25% Omission trial
Tone site nose poke
Reward site nose poke
No pellet
Tone (0.5 s)
Blue light
Yellow light (1 s)
Serotonin no-activation trials
75% reward trial (fixed delay 6 s)
Tone site nose poke
Reward site nose poke
1 pellet
Tone (0.5 s)
Yellow light
Yellow light (1 s)
25% Omission trial
Tone site nose poke
Reward site nose poke
No pellet
Tone (0.5 s)
Yellow light
Yellow light (1 s)
B
Reward delay 4, 6 and 8 s (D4-6-8) test
Serotonin activation trials
75% reward trial (random delay 4, 6 or 8 s)
Tone site nose poke
Reward site nose poke
1 pellet
Tone (0.5 s)
Blue light
Yellow light (1 s)
25% Omission trial
Tone site nose poke
Reward site nose poke
No pellet
Tone (0.5 s)
Blue light
Yellow light (1 s)
Serotonin no-activation trials
75% reward trial (random delay 4, 6 or 8 s)
Tone site nose poke
Reward site nose poke
1 pellet
Tone (0.5 s)
Yellow light
Yellow light (1 s)
25% Omission trial
Tone site nose poke
Reward site nose poke
No pellet
Tone (0.5 s)
Yellow light
Yellow light (1 s)
C
Reward delay 2, 6 and 10 s (D2-6-10) test
Serotonin activation trials
75% reward trial (random delay 2, 6 or 10 s)
Tone site nose poke
Reward site nose poke
1 pellet
Tone (0.5 s)
Blue light
Yellow light (1 s)
25% Omission trial
Tone site nose poke
Reward site nose poke
No pellet
Tone (0.5 s)
Blue light
Yellow light (1 s)
Serotonin no-activation trials
75% reward trial (random delay 2, 6 or 10 s)
Tone site nose poke
Reward site nose poke
1 pellet
Tone (0.5 s)
Yellow light
Yellow light (1 s)
25% Omission trial
Tone site nose poke
Reward site nose poke
No pellet
Tone (0.5 s)
Yellow light
Yellow light (1 s)
D
Reward delay 10 s (D10) test
Serotonin activation trials
75% reward trial (fixed delay 10 s)
Tone site nose poke
Reward site nose poke
1 pellet
Tone (0.5 s)
Blue light
Yellow light (1 s)
25% Omission trial
Tone site nose poke
Reward site nose poke
No pellet
Tone (0.5 s)
Blue light
Yellow light (1 s)
Serotonin no-activation trials
75% reward trial (fixed delay 10 s)
Tone site nose poke
Reward site nose poke
1 pellet
Tone (0.5 s)
Yellow light
Yellow light (1 s)
25% Omission trial
Tone site nose poke
Reward site nose poke
No pellet
Tone (0.5 s)
Yellow light
Yellow light (1 s)

Fig. S2. Schematic of the D6, D4-6-8, D2-6-10 and D10 tests.

(**A**) Time sequence of serotonin activation trials and serotonin no-activation trials in the D6 test. 75% of these trials were rewarded after a 6-s delay. Food pellets were omitted in 25% of these trials. In serotonin activation trials, continuous blue light was delivered from the onset of the reward delay until the onset of food presentation or a reward-wait error. In serotonin no-activation trials, continuous yellow light was applied from the onset of the reward delay until the onset of food presentation or a reward-wait error. In each trial, 1 s of yellow light was used to indicate the onset of food presentation or a reward-wait error. Blue and yellow bars denote blue- and yellow-light stimulation, respectively. Brown- and red-shaded regions denote tone- and reward-delay periods, respectively. Orange-shaded regions denote durations of tone presentation. (**B**) Time sequence of serotonin activation trials and serotonin no-activation trials in the D4-6-8 test. 75% of these trials were rewarded after a 4, 6, or 8-s delay. Food pellets were omitted in 25% of these trials. (**C**) Time sequence of serotonin activation trials and serotonin no-activation trials in the D2-6-10 test. 75% of these trials were rewarded after a 2, 6, or 10-s delay. Food pellets were omitted in 25% of these trials. (**D**) Time sequence of serotonin activation trials and serotonin no-activation trials in the D10 test. 75% of these trials were rewarded after a 10-s delay. Food pellets were omitted in 25% of these trials. Each test lasted until a mouse completed 40 trials. In the D6 and D10 tests, eight trials (three fixed-delay with serotonin activation, one omission with serotonin activation, three fixed-delay without serotonin activation, and one omission without serotonin activation) were randomly selected without repetition until all items were selected, and then this selection was repeated five times. In the D4-6-8 and D2-6-10 tests, the eight trial patterns (two light conditions x four delay lengths) were randomly selected without repetition until all items were selected, and then this selection was repeated five times.

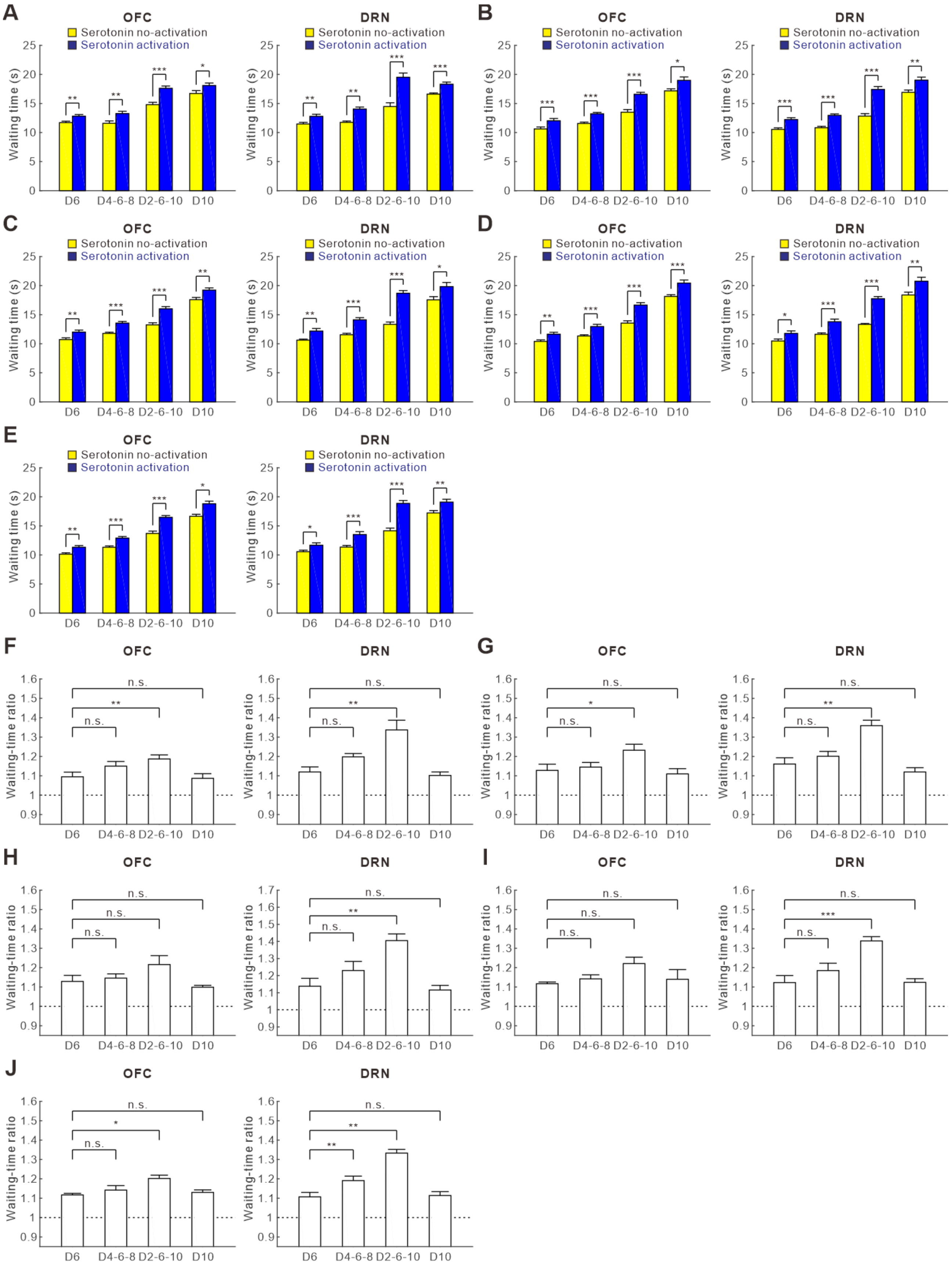

A
B
C
D
E
F
G
H
I
J
OFC
DRN
Serotonin no-activation
Serotonin activation
Waiting time (s)
Waiting-time ratio
D6
D4-6-8
D2-6-10
D10
n.s.

Fig. S3. Waiting times and waiting-time ratios during omission trials for each mouse with implanted optic fibers in both the OFC and DRN.

(**A-E**) Waiting time during omission trials in the D6, D4-6-8, D2-6-10 and D10 tests for each of the five tested mice. (**A**) OFC: D6 test, yellow, $n = 45$, blue, $n = 45$, $U = 628$, $P = 0.0019$; D4-6-8 test, yellow, $n = 35$, blue, $n = 35$, $U = 334$, $P = 0.0011$; D2-6-10 test, yellow, $n = 40$, blue, $n = 40$, $U = 364$, $P = 2.7 \times 10^{-5}$, D10 test, yellow, $n = 28$, blue, $n = 28$, $U = 269$, $P = 0.044$. DRN: D6 test, yellow, $n = 30$, blue, $n = 30$, $U = 265.5$, $P = 0.0064$; D4-6-8 test, yellow, $n = 35$, blue, $n = 35$, $U = 182$, $P < 10^{-6}$; D2-6-10 test, yellow, $n = 30$, blue, $n = 30$, $U = 133.5$, $P < 10^{-6}$, D10 test, yellow, $n = 30$, blue, $n = 30$, $U = 172.5$, $P = 4.1 \times 10^{-5}$. (**B**) OFC: D6 test, yellow, $n = 33$, blue, $n = 35$, $U = 295$, $P = 5.3 \times 10^{-4}$; D4-6-8 test, yellow, $n = 40$, blue, $n = 40$, $U = 342$, $P = 1.0 \times 10^{-5}$; D2-6-10 test, yellow, $n = 35$, blue, $n = 35$, $U = 208.5$, $P < 10^{-5}$, D10 test, yellow, $n = 29$, blue, $n = 30$, $U = 295$, $P = 0.034$. DRN: D6 test, yellow, $n = 30$, blue, $n = 29$, $U = 187.5$, $P = 1.8 \times 10^{-4}$; D4-6-8 test, yellow, $n = 35$, blue, $n = 35$, $U = 175.5$, $P < 10^{-6}$; D2-6-10 test, yellow, $n = 30$, blue, $n = 30$, $U = 55$, $P < 10^{-6}$, D10 test, yellow, $n = 33$, blue, $n = 35$, $U = 339$, $P = 0.0034$. (**C**) OFC: D6 test, yellow, $n = 40$, blue, $n = 40$, $U = 497$, $P = 0.0035$; D4-6-8 test, yellow, $n = 35$, blue, $n = 35$, $U = 196.5$, $P < 10^{-5}$; D2-6-10 test, yellow, $n = 40$, blue, $n = 40$, $U = 311$, $P < 10^{-5}$, D10 test, yellow, $n = 38$, blue, $n = 40$, $U = 489.5$, $P = 0.0068$. DRN: D6 test, yellow, $n = 35$, blue, $n = 34$, $U = 366$, $P = 0.0060$; D4-6-8 test, yellow, $n = 40$, blue, $n = 40$, $U = 230.5$, $P < 10^{-6}$; D2-6-10 test, yellow, $n = 40$, blue, $n = 40$, $U = 130$, $P < 10^{-6}$, D10 test, yellow, $n = 40$, blue, $n = 40$, $U = 555$, $P = 0.018$. (**D**) OFC: D6 test, yellow, $n = 35$, blue, $n = 35$, $U = 362$, $P = 0.0033$; D4-6-8 test, yellow, $n = 29$, blue, $n = 30$, $U = 198$, $P = 3.3 \times 10^{-4}$; D2-6-10 test, yellow, $n = 40$, blue, $n = 40$, $U = 310.5$, $P < 10^{-5}$, D10 test, yellow, $n = 35$, blue, $n = 35$, $U = 324$, $P = 7.0 \times 10^{-4}$. DRN: D6 test, yellow, $n = 30$, blue, $n = 30$, $U = 283$, $P = 0.014$; D4-6-8 test, yellow, $n = 30$, blue, $n = 30$, $U = 157.5$, $P < 10^{-6}$; D2-6-10 test, yellow, $n = 45$, blue, $n = 45$, $U = 163.5$, $P < 10^{-6}$, D10 test, yellow, $n = 35$, blue, $n = 35$, $U = 383$, $P = 0.0070$. (**E**) OFC: D6 test, yellow, $n = 30$, blue, $n = 30$, $U = 253.5$, $P = 0.0037$; D4-6-8 test, yellow, $n = 35$, blue, $n = 35$, $U = 242.5$, $P = 1.4 \times 10^{-5}$; D2-6-10 test, yellow, $n = 35$, blue, $n = 35$, $U = 212$, $P < 10^{-5}$, D10 test, yellow, $n = 30$, blue, $n = 30$, $U = 232$, $P = 0.013$. DRN: D6 test, yellow, $n = 30$, blue, $n = 30$, $U = 310.5$, $P = 0.039$; D4-6-8 test, yellow, $n = 30$, blue, $n = 30$, $U = 171.5$, $P < 10^{-6}$; D2-6-10 test, yellow, $n = 34$, blue, $n = 35$, $U = 119.5$, $P < 10^{-6}$, D10 test, yellow, $n = 30$, blue, $n = 29$, $U = 261.5$, $P = 0.0085$. $^{*}P < 0.05$, $^{**}P < 0.01$, $^{***}P < 0.001$ by Mann-Whitney U test. Error bars represent the s.e.m.

(**F-J**) Waiting time ratios in the D6, D4-6-8, D2-6-10 and D10 tests for each of the five tested mice. (**F**) OFC: D6 test, $n = 9$; D4-6-8 test, $n = 7$; D2-6-10 test, $n = 8$; D10 test, $n = 7$; D6 vs. D4-6-8, $U = 15$, $P = 0.091$; D6 vs. D2-6-10, $U = 7$, $P = 0.0037$; D6 vs. D10, $U= 27$, $P = 0.68$. DRN: D6 test, $n = 6$; D4-6-8 test, $n = 7$; D2-6-10 test, $n = 6$; D10 test, $n = 6$; D6 vs. D4-6-8, $U = 8$, $P = 0.073$; D6 vs. D2-6-10, $U = 0$, $P = 0.0022$; D6 vs. D10, $U= 16$, $P = 0.82$. (**G**) OFC: D6 test, $n = 7$; D4-6-8 test, $n = 8$; D2-6-10 test, $n = 7$; D10 test, $n = 6$; D6 vs. D4-6-8, $U = 25$, $P = 0.78$; D6 vs. D2-6-10, $U = 8$, $P = 0.038$; D6 vs. D10, $U= 16$, $P = 0.53$. DRN: D6 test, $n = 6$; D4-6-8 test, $n = 7$; D2-6-10 test, $n = 6$; D10 test, $n = 7$; D6 vs. D4-6-8, $U = 13$, $P = 0.30$; D6 vs. D2-6-10, $U = 0$, $P = 0.0022$; D6 vs. D10, $U= 13$, $P = 0.30$. (**H**) OFC: D6 test, $n = 8$; D4-6-8 test, $n = 7$; D2-6-10 test, $n = 8$; D10 test, $n = 8$; D6 vs. D4-6-8, $U = 22$, $P = 0.54$; D6 vs. D2-6-10, $U = 14$, $P = 0.065$; D6 vs. D10, $U= 29.5$, $P = 0.80$. DRN: D6 test, $n = 7$; D4-6-8 test, $n = 8$; D2-6-10 test, $n = 8$; D10 test, $n = 8$; D6 vs. D4-6-8, $U = 15$, $P = 0.15$; D6 vs. D2-6-10, $U = 2$, $P = 0.0012$; D6 vs. D10, $U= 27$, $P = 0.96$. (**I**) OFC: D6 test, $n = 7$; D4-6-8 test, $n = 6$; D2-6-10 test, $n = 8$; D10 test, $n = 7$; D6 vs. D4-6-8, $U = 15.5$, $P = 0.45$; D6 vs. D2-6-10, $U = 11.5$, $P = 0.054$; D6 vs. D10, $U= 24$, $P = 1.00$. DRN: D6 test, $n = 6$; D4-6-8 test, $n = 6$; D2-6-10 test, $n = 9$; D10 test, $n = 7$; D6 vs. D4-6-8, $U = 12$, $P = 0.39$; D6 vs. D2-6-10, $U = 0.5$, $P = 4.0 \times 10^{-4}$; D6 vs. D10, $U= 20$, $P = 0.95$. (**J**) OFC: D6 test, $n = 6$; D4-6-8 test, $n = 7$; D2-6-10 test, $n = 7$; D10 test, $n = 6$; D6 vs. D4-6-8, $U = 13.5$, $P = 0.30$; D6 vs. D2-6-10, $U = 3$, $P = 0.014$; D6 vs. D10, $U= 15$, $P = 0.70$. DRN: D6 test, $n = 6$; D4-6-8 test, $n = 6$; D2-6-10 test, $n = 7$; D10 test, $n = 6$; D6 vs. D4-6-8, $U = 1.5$, $P = 0.0043$; D6 vs. D2-6-10, $U = 0$, $P = 0.0012$; D6 vs. D10, $U= 16.5$, $P = 0.82$. $^{*}P < 0.05$, $^{**}P < 0.01$, $^{***}P < 0.001$ by Mann-Whitney U test. n.s., not significant. Error bars represent the s.e.m.

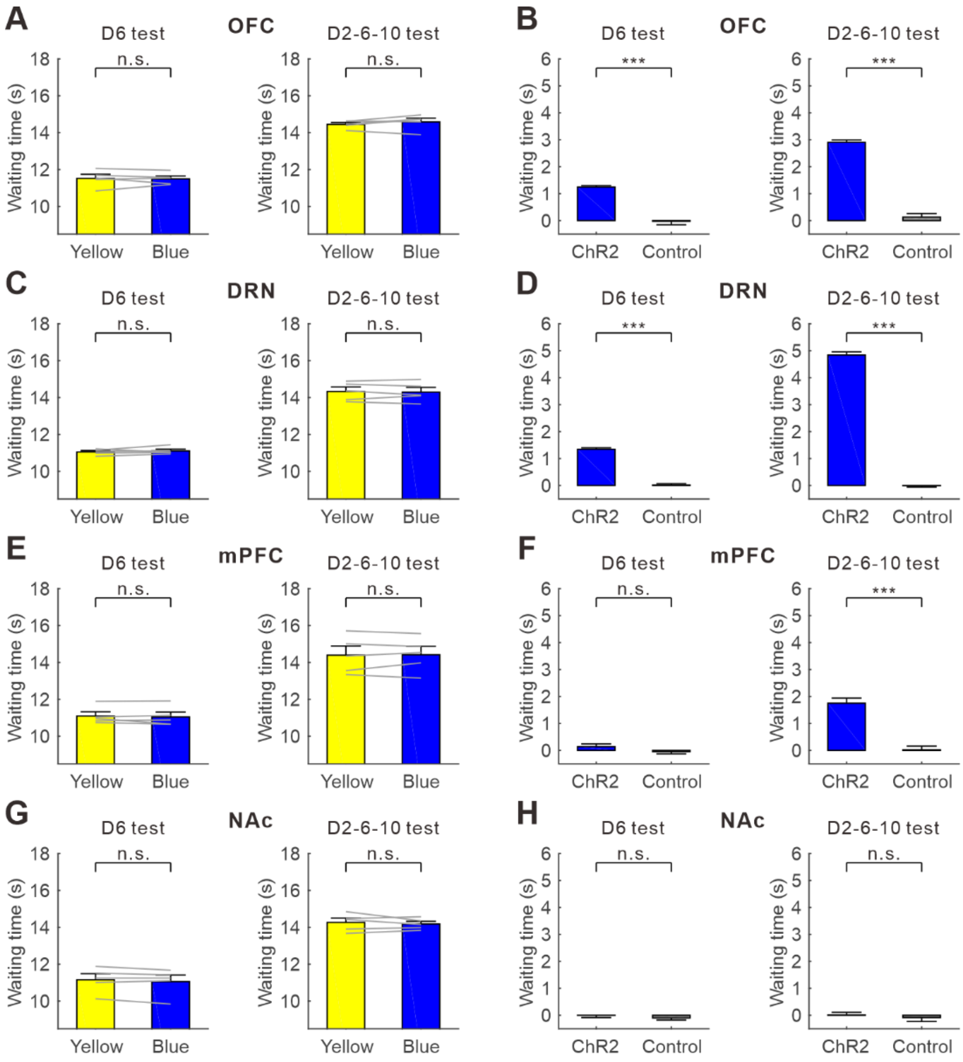

Fig. S4. Waiting times during omission trials for control mice.

(**A**) Average waiting time with yellow- and blue-light stimulation of the OFC during D6 and D2-6-10 tests. Grey lines indicate waiting times for individual control mice (n = 5). n.s., not significant. Error bars represent the s.e.m. (**B**) Difference of waiting times between OFC yellow- and blue-light stimulation of transgenic mice (ChR2, n = 5) and wild-type mice (Control, n = 5). ***P < 0.001 by unpaired t-test. Error bars represent the s.e.m. (**C**) Average waiting time with yellow- and blue-light stimulation of the DRN during D6 and D2-6-10 tests. Grey lines indicate waiting times for individual control mice (n = 5). n.s., not significant. Error bars represent the s.e.m. (**D**) Difference of waiting times between DRN yellow- and blue-light stimulation of transgenic mice (ChR2, n = 15) and wild-type mice (Control, n = 5). ***P < 0.001 by unpaired t-test. Error bars represent the s.e.m. (**E**) Average waiting time with yellow- and blue-light stimulation of the mPFC during D6 and D2-6-10 tests. Grey lines indicate waiting times for individual control mice (n = 5). n.s., not significant. Error bars represent the s.e.m. (**F**) Difference of waiting times between mPFC yellow- and blue-light stimulation of transgenic mice (ChR2, n = 5) and wild-type mice (Control, n = 5). ***P < 0.001 by unpaired t-test. n.s., not significant. Error bars represent the s.e.m. (**G**) Average waiting time with yellow- and blue-light stimulation of the OFC during D6 and D2-6-10 tests. Grey lines indicate waiting times for individual control mice (n = 5). n.s., not significant. Error bars represent the s.e.m. (**H**) Difference of waiting times between NAc yellow- and blue-light stimulation of transgenic mice (ChR2, n = 5) and wild-type mice (Control, n = 5). n.s., not significant. Error bars represent the s.e.m.

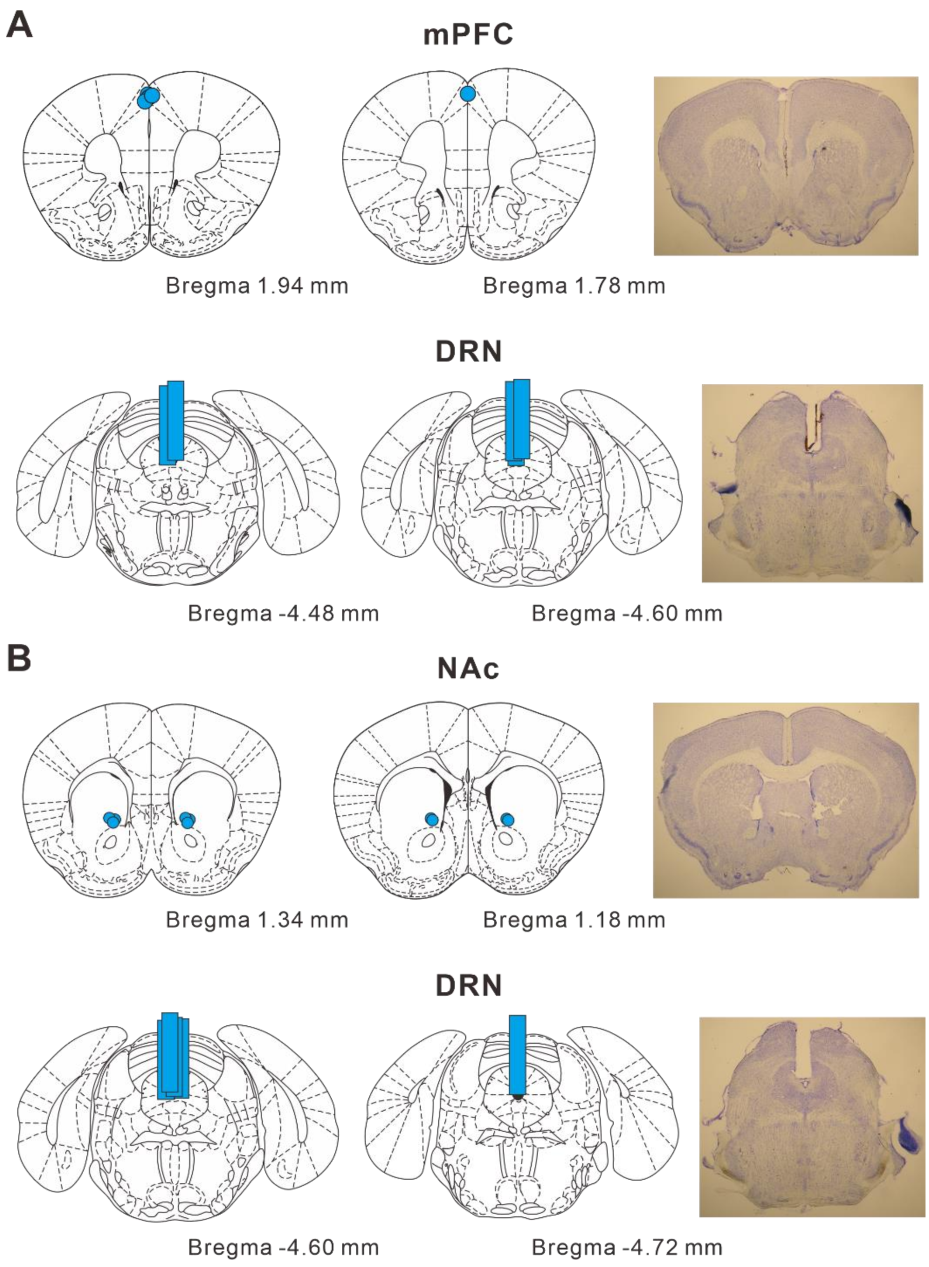

Fig. S5. Locations of optic fibers.
(**A**) Locations of optic fibers in the mPFC and DRN and representative fiber trace for mPFC and DRN. Light blue circles in the mPFC represent tip positions of optic fibers. Light blue bars in the DRN represent tracks of implanted optic fibers. Coronal drawings were adapted from ref. (*54*) with permission. (**B**) Locations of optic fibers in the NAc and DRN representative fiber trace for NAc and DRN. Light blue circles in the NAc represent tip positions of optic fibers. Light blue bars in the DRN represent tracks of implanted optic fibers. Coronal drawings were adapted from ref. (*54*) with permission.

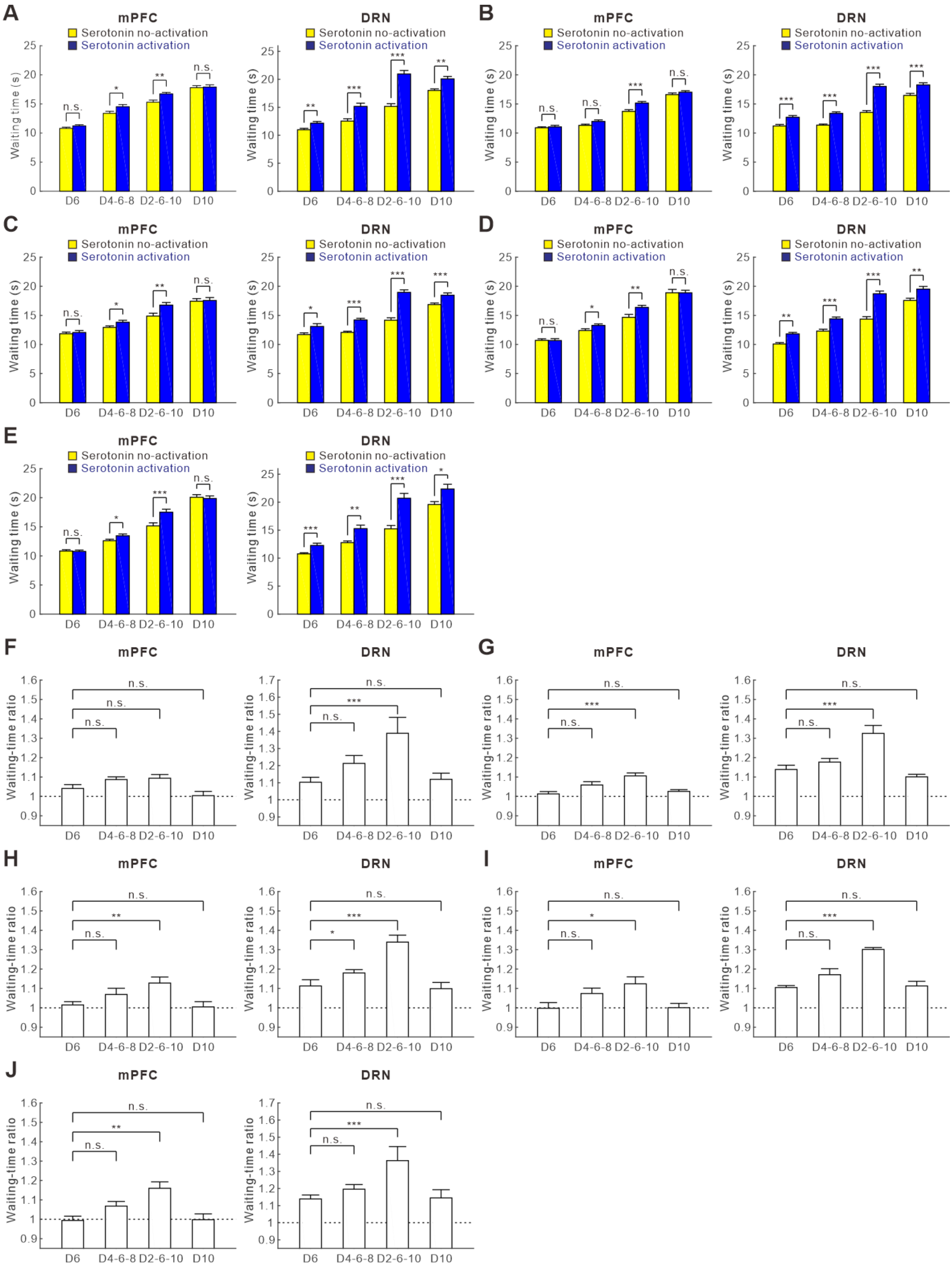
A
B
C
D
E
F
G
H
I
J
mPFC
DRN
Serotonin no-activation
Serotonin activation
Waiting time (s)
Waiting-time ratio
D6
D4-6-8
D2-6-10
D10
n.s.
*
**
***

Fig. S6. Waiting times and waiting-time ratios during omission trials for each mouse with implanted optic fibers in both the mPFC and DRN.

(**A-E**) Waiting time during omission trials in the D6, D4-6-8, D2-6-10 and D10 tests for each of the five tested mice. (**A**) mPFC: D6 test, yellow, n = 50, blue, n = 50, U = 978.5, P = 0.061; D4-6-8 test, yellow, n = 47, blue, n = 50, U = 777.5, P = 0.015; D2-6-10 test, yellow, n = 50, blue, n = 50, U = 823.5, P = 0.0033, D10 test, yellow, n = 45, blue, n = 45, U = 984, P = 0.82. DRN: D6 test, yellow, n = 40, blue, n = 39, U = 498, P = 0.0057; D4-6-8 test, yellow, n = 30, blue, n = 30, U = 184.5, $P = 8.7 \times 10^{-5}$; D2-6-10 test, yellow, n = 45, blue, n = 44, U = 168.5, $P < 10^{-6}$, D10 test, yellow, n = 45, blue, n = 45, U = 608.5, P = 0.0011. (**B**) mPFC: D6 test, yellow, n = 40, blue, n = 40, U = 777, P = 0.83; D4-6-8 test, yellow, n = 40, blue, n = 40, U = 609, P = 0.066; D2-6-10 test, yellow, n = 50, blue, n = 50, U = 756, $P = 6.6 \times 10^{-4}$, D10 test, yellow, n = 45, blue, n = 44, U = 855, P = 0.27. DRN: D6 test, yellow, n = 50, blue, n = 50, U = 711.5, $P = 2.1 \times 10^{-4}$; D4-6-8 test, yellow, n = 45, blue, n = 44, U = 313.5, $P < 10^{-6}$; D2-6-10 test, yellow, n = 50, blue, n = 50, U = 207.5, $P < 10^{-6}$, D10 test, yellow, n = 40, blue, n = 40, U = 425, $P = 3.1 \times 10^{-4}$. (**C**) mPFC: D6 test, yellow, n = 50, blue, n = 50, U = 1197, P = 0.72; D4-6-8 test, yellow, n = 40, blue, n = 40, U = 591, P = 0.044; D2-6-10 test, yellow, n = 40, blue, n = 40, U = 479.5, P = 0.0020, D10 test, yellow, n = 38, blue, n = 40, U = 758.5, P = 0.99. DRN: D6 test, yellow, n = 50, blue, n = 49, U = 920, P = 0.033; D4-6-8 test, yellow, n = 45, blue, n = 45, U = 303.5, $P < 10^{-6}$; D2-6-10 test, yellow, n = 45, blue, n = 45, U = 208.5, $P < 10^{-6}$, D10 test, yellow, n = 45, blue, n = 45, U = 598, $P = 8.2 \times 10^{-4}$. (**D**) mPFC: D6 test, yellow, n = 35, blue, n = 35, U = 593.5, P = 0.82; D4-6-8 test, yellow, n = 35, blue, n = 35, U = 418.5, P = 0.023; D2-6-10 test, yellow, n = 35, blue, n = 35, U = 384, P = 0.0073, D10 test, yellow, n = 30, blue, n = 30, U = 410.5, P = 0.56. DRN: D6 test, yellow, n = 35, blue, n = 35, U = 348, P = 0.0019; D4-6-8 test, yellow, n = 34, blue, n = 34, U = 260.5, $P = 9.8 \times 10^{-6}$; D2-6-10 test, yellow, n = 45, blue, n = 45, U = 292, $P < 10^{-6}$, D10 test, yellow, n = 30, blue, n = 30, U = 247.5, P = 0.0028. (**E**) mPFC: D6 test, yellow, n = 35, blue, n = 35, U = 559, P = 0.53; D4-6-8 test, yellow, n = 35, blue, n = 35, U = 444, P = 0.048; D2-6-10 test, yellow, n = 35, blue, n = 35, U = 308, $P = 3.5 \times 10^{-4}$, D10 test, yellow, n = 30, blue, n = 30, U = 448, P = 0.98. DRN: D6 test, yellow, n = 30, blue, n = 30, U = 185, $P = 8.9 \times 10^{-5}$; D4-6-8 test, yellow, n = 30, blue, n = 30, U = 241, P = 0.0020; D2-6-10 test, yellow, n = 35, blue, n = 35, U = 156, $P < 10^{-6}$, D10 test, yellow, n = 28, blue, n = 30, U = 260.5, P = 0.013. *$P < 0.05$, **$P < 0.01$, ***$P < 0.001$ by Mann-Whitney U test. Error bars represent the s.e.m. (**F-J**) Waiting time ratios in the D6, D4-6-8, D2-6-10 and D10 tests for each of the five tested mice. (**F**) mPFC: D6 test, n = 10; D4-6-8 test, n = 10; D2-6-10 test, n = 10; D10 test, n = 9; D6 vs. D4-6-8, U = 31, P = 0.17; D6 vs. D2-6-10, U = 30.5, P = 0.14; D6 vs. D10, U= 30, P = 0.24. DRN: D6 test, n = 8; D4-6-8 test, n = 6; D2-6-10 test, n =9; D10 test, n = 9; D6 vs. D4-6-8, U = 12, P = 0.14; D6 vs. D2-6-10, U = 4, $P = 9.9 \times 10^{-4}$; D6 vs. D10, U= 34, P = 0.89. (**G**) mPFC: D6 test, n = 8; D4-6-8 test, n = 8; D2-6-10 test, n = 10; D10 test, n = 9; D6 vs. D4-6-8, U = 12, P = 0.038; D6 vs. D2-6-10, U = 4, $P = 5.5 \times 10^{-4}$; D6 vs. D10, U= 25, P = 0.32. DRN: D6 test, n = 10; D4-6-8 test, n = 9; D2-6-10 test, n = 10; D10 test, n = 8; D6 vs. D4-6-8, U = 27, P = 0.16; D6 vs. D2-6-10, U = 4.5, $P = 1.3 \times 10^{-4}$; D6 vs. D10, U= 21, P = 0.10. (**H**) mPFC: D6 test, n = 10; D4-6-8 test, n = 8; D2-6-10 test, n = 8; D10 test, n = 8; D6 vs. D4-6-8, U = 22, P = 0.12; D6 vs. D2-6-10, U = 8, P = 0.0031; D6 vs. D10, U= 36, P = 0.76. DRN: D6 test, n = 10; D4-6-8 test, n = 9; D2-6-10 test, n = 9; D10 test, n = 9; D6 vs. D4-6-8, U = 20, P = 0.043; D6 vs. D2-6-10, U = 5.5, $P = 4.1 \times 10^{-4}$; D6 vs. D10, U= 38.5, P = 0.60. (**I**) mPFC: D6 test, n = 7; D4-6-8 test, n = 7; D2-6-10 test, n = 7; D10 test, n =6; D6 vs. D4-6-8, U = 9, P = 0.053; D6 vs. D2-6-10, U = 5, P = 0.011; D6 vs. D10, U= 20, P = 0.95. DRN: D6 test, n = 7; D4-6-8 test, n = 7; D2-6-10 test, n = 9; D10 test, n = 6; D6 vs. D4-6-8, U = 11, P = 0.097; D6 vs. D2-6-10, U = 0, $P = 1.8 \times 10^{-4}$; D6 vs. D10, U= 20, P = 0.95. (**J**) mPFC: D6 test, n = 7; D4-6-8 test, n = 7; D2-6-10 test, n = 7; D10 test, n = 6; D6 vs. D4-6-8, U = 9, P = 0.053; D6 vs. D2-6-10, U = 1, P = 0.0012; D6 vs. D10, U= 17, P = 0.63. DRN: D6 test, n = 6; D4-6-8 test, n = 6; D2-6-10 test, n = 7; D10 test, n = 6; D6 vs. D4-6-8, U = 10, P = 0.24; D6 vs. D2-6-10, U = 2, P = 0.0047; D6 vs. D10, U= 14, P = 0.59. *$P < 0.05$, **$P < 0.01$, ***$P < 0.001$ by Mann-Whitney U test. n.s., not significant. Error bars represent the s.e.m.

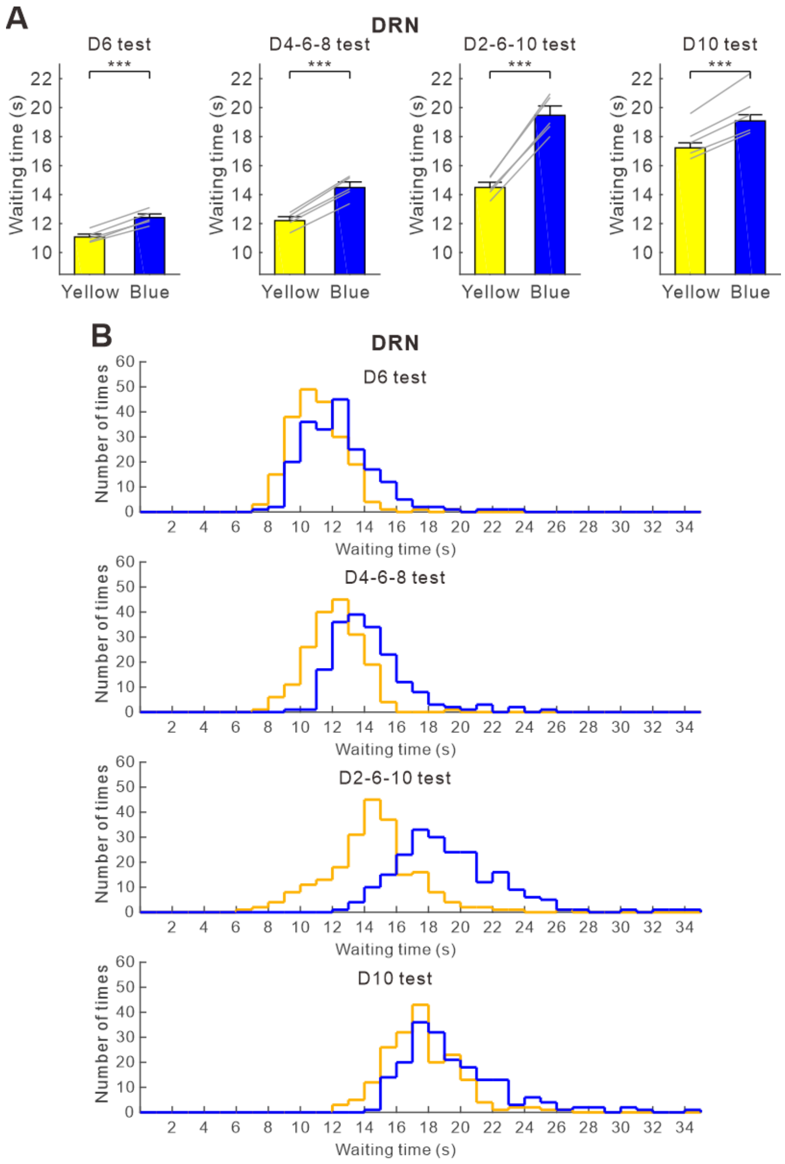

Fig. S7. Effect of DRN photostimulation on waiting time with implanted optic fibers in both the mPFC and DRN.

(**A**) Average waiting times in no-activation (yellow) and activation (blue) of DRN serotonin neurons during the four reward-delay tests. Grey lines indicate waiting times for individual ChR2-expressing mice (n = 5). (**B**) Distribution of waiting times during omission trials in no-activation (yellow) and activation (blue) of DRN serotonergic neurons during the four reward-delay tests. ***P < 0.001 by paired t-test. Error bars represent the s.e.m.

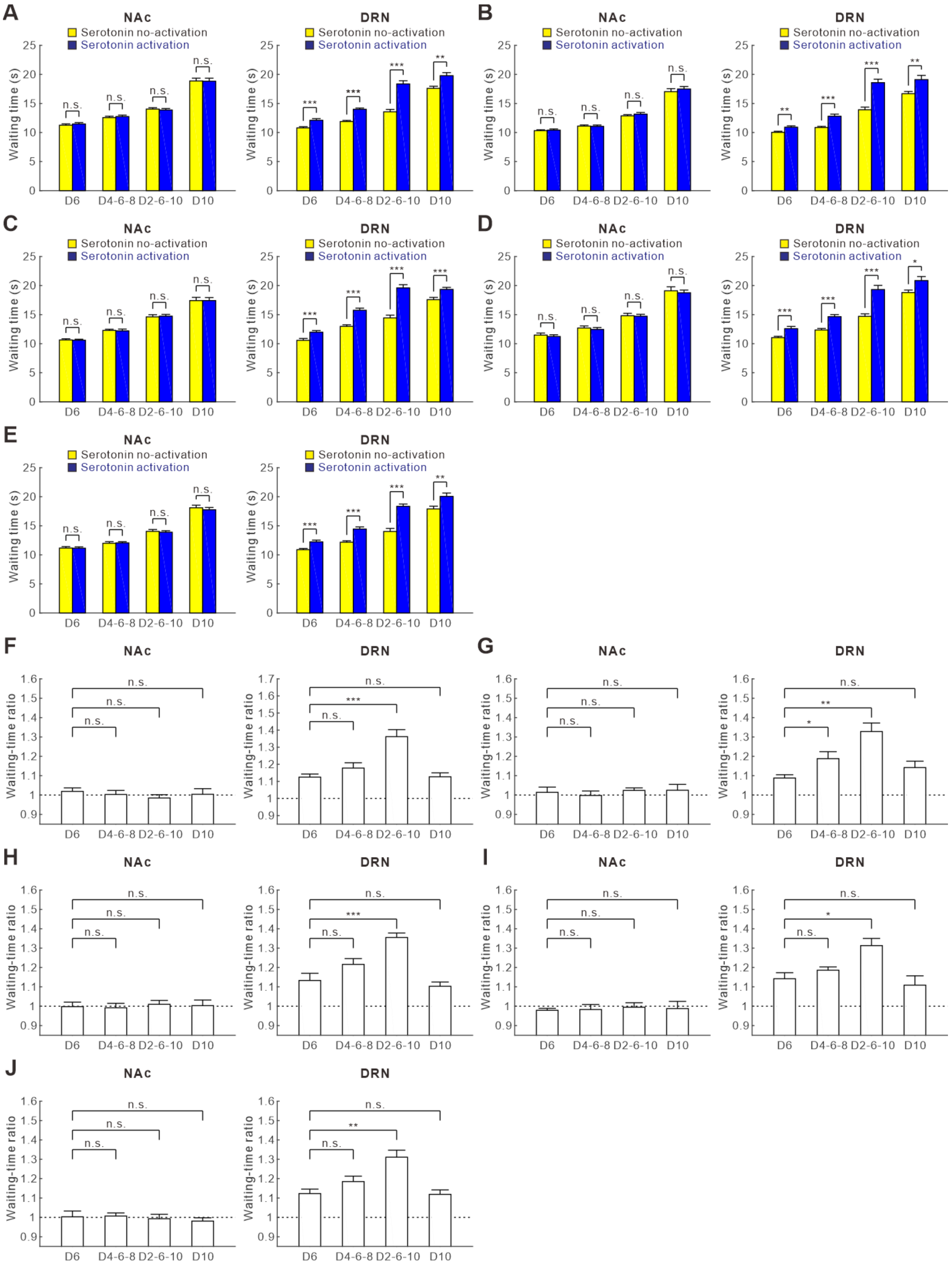
A
NAc
Serotonin no-activation
Serotonin activation
Waiting time (s)
25
20
15
10
5
0
n.s.
D6
D4-6-8
D2-6-10
D10
DRN
***
**
B
NAc
DRN
C
NAc
DRN
D
NAc
DRN
*
E
NAc
DRN
F
NAc
DRN
Waiting-time ratio
1.7
1.6
1.5
1.4
1.3
1.2
1.1
1
0.9
G
NAc
DRN
H
NAc
DRN
I
NAc
DRN
J
NAc
DRN

Fig. S8. Waiting times and waiting-time ratios during omission trials for each mouse with implanted optic fibers in both the NAc and DRN.

(**A-E**) Waiting time during omission trials in the D6, D4-6-8, D2-6-10 and D10 tests for each of the five tested mice. (**A**) NAc: D6 test, yellow, $n = 54$, blue, $n = 55$, $U = 1383.5$, $P = 0.54$; D4-6-8 test, yellow, $n = 33$, blue, $n = 34$, $U = 528.5$, $P = 0.68$; D2-6-10 test, yellow, $n = 35$, blue, $n = 35$, $U = 569$, $P = 0.61$, D10 test, yellow, $n = 39$, blue, $n = 39$, $U = 733$, $P = 0.78$. DRN: D6 test, yellow, $n = 55$, blue, $n = 54$, $U = 904.5$, $P = 4.3 \times 10^{-4}$; D4-6-8 test, yellow, $n = 45$, blue, $n = 45$, $U = 262$, $P < 10^{-6}$; D2-6-10 test, yellow, $n = 40$, blue, $n = 39$, $U = 151.5$, $P < 10^{-6}$, D10 test, yellow, $n = 27$, blue, $n = 28$, $U = 197$, $P = 0.0023$. (**B**) NAc: D6 test, yellow, $n = 29$, blue, $n = 30$, $U = 376.5$, $P = 0.38$; D4-6-8 test, yellow, $n = 35$, blue, $n = 34$, $U = 569$, $P = 0.76$; D2-6-10 test, yellow, $n = 50$, blue, $n = 50$, $U = 1061$, $P = 0.19$, D10 test, yellow, $n = 35$, blue, $n = 35$, $U = 526$, $P = 0.31$. DRN: D6 test, yellow, $n = 25$, blue, $n = 25$, $U = 177.5$, $P = 0.0088$; D4-6-8 test, yellow, $n = 35$, blue, $n = 34$, $U = 210.5$, $P < 10^{-5}$; D2-6-10 test, yellow, $n = 40$, blue, $n = 40$, $U = 199$, $P < 10^{-6}$, D10 test, yellow, $n = 28$, blue, $n = 27$, $U = 226$, $P = 0.010$. (**C**) NAc: D6 test, yellow, $n = 40$, blue, $n = 40$, $U = 794.5$, $P = 0.96$; D4-6-8 test, yellow, $n = 35$, blue, $n = 35$, $U = 581.5$, $P = 0.72$; D2-6-10 test, yellow, $n = 45$, blue, $n = 45$, $U = 964.5$, $P = 0.70$, D10 test, yellow, $n = 40$, blue, $n = 40$, $U = 774$, $P = 0.80$. DRN: D6 test, yellow, $n = 40$, blue, $n = 40$, $U = 417$, $P = 2.3 \times 10^{-4}$; D4-6-8 test, yellow, $n = 35$, blue, $n = 35$, $U = 176$, $P < 10^{-6}$; D2-6-10 test, yellow, $n = 45$, blue, $n = 45$, $U = 242.5$, $P < 10^{-6}$, D10 test, yellow, $n = 40$, blue, $n = 40$, $U = 454.5$, $P = 8.9 \times 10^{-4}$. (**D**) NAc: D6 test, yellow, $n = 30$, blue, $n = 30$, $U = 435$, $P = 0.82$; D4-6-8 test, yellow, $n = 30$, blue, $n = 30$, $U = 414.5$, $P = 0.60$; D2-6-10 test, yellow, $n = 30$, blue, $n = 30$, $U = 440$, $P = 0.88$, D10 test, yellow, $n = 30$, blue, $n = 30$, $U = 440$, $P = 0.88$. DRN: D6 test, yellow, $n = 30$, blue, $n = 30$, $U = 215$, $P = 5.1 \times 10^{-4}$; D4-6-8 test, yellow, $n = 35$, blue, $n = 35$, $U = 196$, $P < 10^{-5}$; D2-6-10 test, yellow, $n = 30$, blue, $n = 30$, $U = 97$, $P < 10^{-6}$, D10 test, yellow, $n = 30$, blue, $n = 30$, $U = 303.5$, $P = 0.030$. (**E**) NAc: D6 test, yellow, $n = 30$, blue, $n = 30$, $U = 436$, $P = 0.84$; D4-6-8 test, yellow, $n = 35$, blue, $n = 35$, $U = 533$, $P = 0.35$; D2-6-10 test, yellow, $n = 30$, blue, $n = 30$, $U = 426.5$, $P = 0.73$, D10 test, yellow, $n = 35$, blue, $n = 35$, $U = 569$, $P = 0.61$. DRN: D6 test, yellow, $n = 30$, blue, $n = 30$, $U = 194.5$, $P = 1.6 \times 10^{-4}$; D4-6-8 test, yellow, $n = 30$, blue, $n = 30$, $U = 138.5$, $P < 10^{-5}$; D2-6-10 test, yellow, $n = 30$, blue, $n = 30$, $U = 101$, $P < 10^{-6}$, D10 test, yellow, $n = 35$, blue, $n = 35$, $U = 360$, $P = 0.0030$. *$P < 0.05$, **$P < 0.01$, ***$P < 0.001$ by Mann-Whitney U test. Error bars represent the s.e.m. (**F-J**) Waiting time ratios in the D6, D4-6-8, D2-6-10 and D10 tests for each of the five tested mice. (**F**) NAc: D6 test, $n = 11$; D4-6-8 test, $n = 7$; D2-6-10 test, $n = 10$; D10 test, $n = 8$; D6 vs. D4-6-8, $U = 33$, $P = 0.72$; D6 vs. D2-6-10, $U = 43$, $P = 0.43$; D6 vs. D10, $U= 38$, $P = 0.66$. DRN: D6 test, $n = 11$; D4-6-8 test, $n = 9$; D2-6-10 test, $n =8$; D10 test, $n = 6$; D6 vs. D4-6-8, $U = 32.5$, $P = 0.20$; D6 vs. D2-6-10, $U = 2$, $P = 1.1 \times 10^{-4}$; D6 vs. D10, $U= 31$, $P = 0.88$. (**G**) NAc: D6 test, $n = 6$; D4-6-8 test, $n = 7$; D2-6-10 test, $n = 10$; D10 test, $n = 7$; D6 vs. D4-6-8, $U = 18$, $P = 0.73$; D6 vs. D2-6-10, $U = 22$, $P = 0.43$; D6 vs. D10, $U= 21$, $P = 1.00$. DRN: D6 test, $n = 5$; D4-6-8 test, $n = 7$; D2-6-10 test, $n = 8$; D10 test, $n = 6$; D6 vs. D4-6-8, $U = 5$, $P = 0.048$; D6 vs. D2-6-10, $U = 0$, $P = 0.0016$; D6 vs. D10, $U= 8$, $P = 0.25$. (**H**) NAc: D6 test, $n = 8$; D4-6-8 test, $n = 7$; D2-6-10 test, $n = 9$; D10 test, $n = 9$; D6 vs. D4-6-8, $U = 28$, $P = 1.00$; D6 vs. D2-6-10, $U = 33$, $P = 0.82$; D6 vs. D10, $U= 30.5$, $P = 0.88$. DRN: D6 test, $n = 8$; D4-6-8 test, $n = 7$; D2-6-10 test, $n = 9$; D10 test, $n = 8$; D6 vs. D4-6-8, $U = 12$, $P = 0.072$; D6 vs. D2-6-10, $U = 0.5$, $P = 8.2 \times 10^{-5}$; D6 vs. D10, $U= 29.5$, $P = 0.80$. (**I**) NAc: D6 test, $n = 6$; D4-6-8 test, $n = 6$; D2-6-10 test, $n = 6$; D10 test, $n =6$; D6 vs. D4-6-8, $U = 13$, $P = 0.49$; D6 vs. D2-6-10, $U = 16$, $P = 0.82$; D6 vs. D10, $U= 16$, $P = 0.82$. DRN: D6 test, $n = 6$; D4-6-8 test, $n = 7$; D2-6-10 test, $n = 6$; D10 test, $n = 6$; D6 vs. D4-6-8, $U = 8.5$, $P = 0.073$; D6 vs. D2-6-10, $U = 3$, $P = 0.015$; D6 vs. D10, $U= 11$, $P = 0.31$. (**J**) NAc: D6 test, $n = 6$; D4-6-8 test, $n = 7$; D2-6-10 test, $n = 6$; D10 test, $n = 7$; D6 vs. D4-6-8, $U = 19$, $P = 0.84$; D6 vs. D2-6-10, $U = 18$, $P = 1.00$; D6 vs. D10, $U= 17.5$, $P = 0.63$. DRN: D6 test, $n = 6$; D4-6-8 test, $n = 6$; D2-6-10 test, $n = 6$; D10 test, $n = 7$; D6 vs. D4-6-8, $U = 6.5$, $P = 0.65$; D6 vs. D2-6-10, $U = 0$, $P = 0.0022$; D6 vs. D10, $U= 18.5$, $P = 0.73$. *$P < 0.05$, **$P < 0.01$, ***$P < 0.001$ by Mann-Whitney U test. n.s., not significant. Error bars represent the s.e.m.

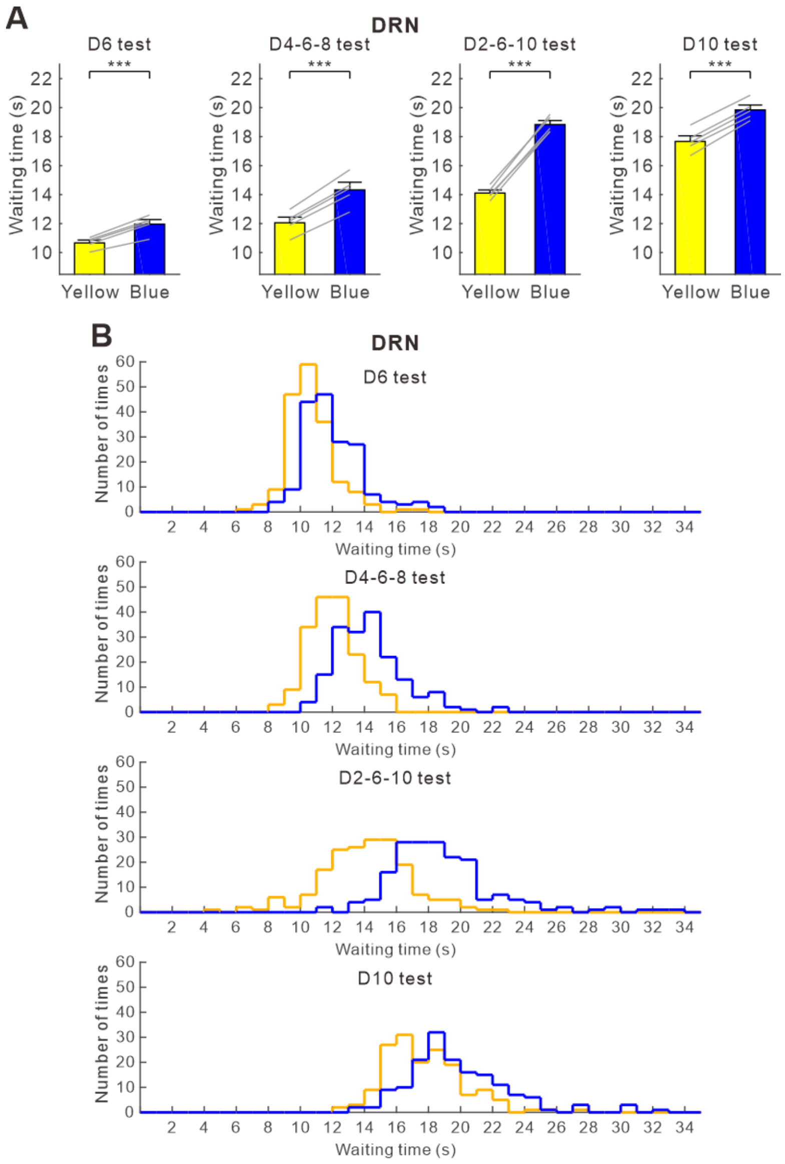

Fig. S9. Effect of DRN photostimulation on waiting times in mice with implanted optic fibers in both the NAc and DRN.

(**A**) Average waiting times in no-activation (yellow) and activation (blue) of DRN serotonergic neurons during the four reward-delay tests. Grey lines indicate waiting times for individual ChR2-expressing mice (*n* = 5). (**B**) Distribution of waiting times during omission trials in no-activation (yellow) and activation (blue) of DRN serotonergic neurons during the four reward-delay tests. ***$P < 0.001$ by paired *t*-test. Error bars represent the s.e.m.

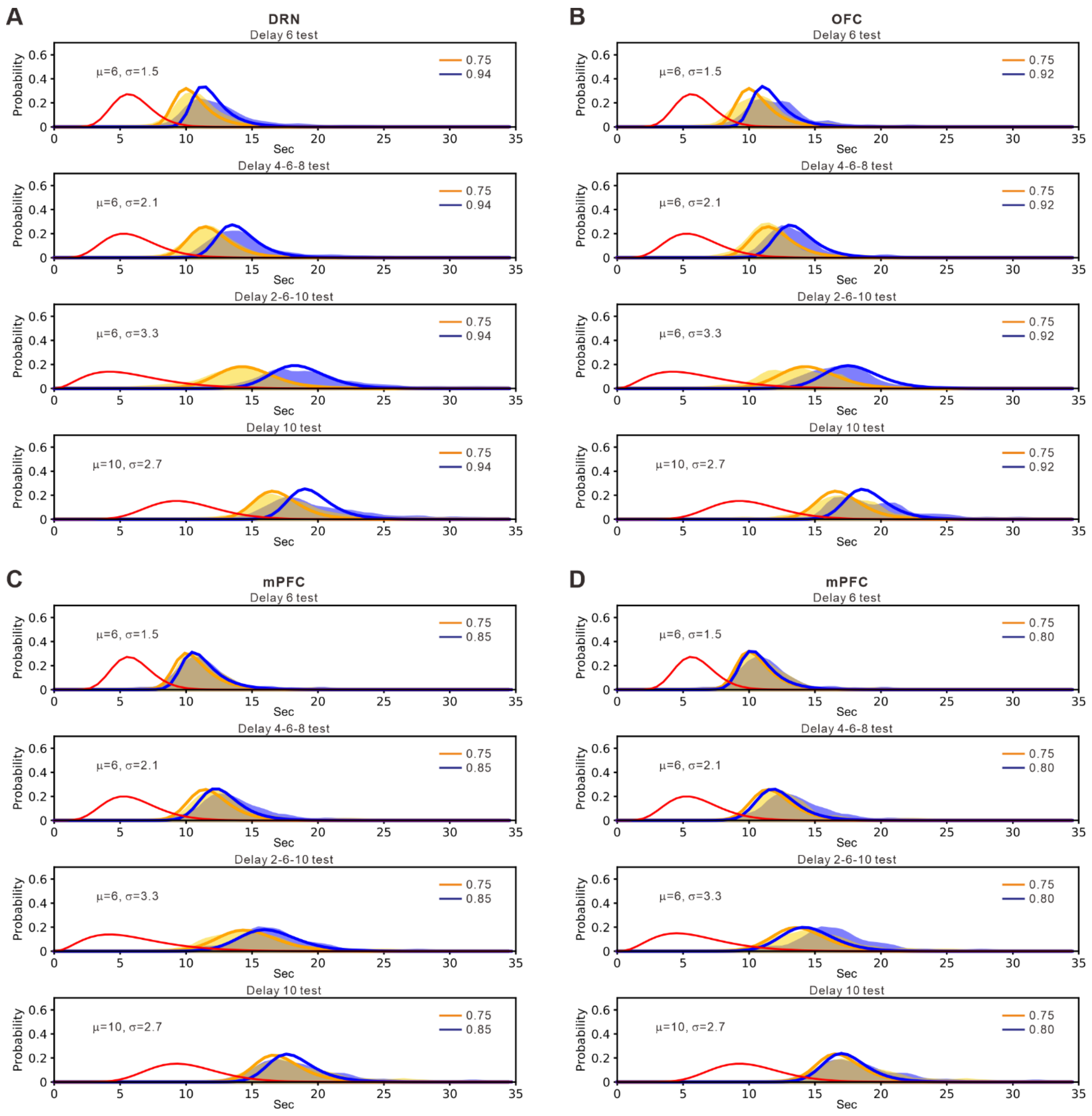

Fig. S10. The assumption that DRN, OFC, and mPFC photostimulation induce different prior probability shifts does not explain behavioral data.

(**A**) If a DRN photostimulation shift prior probability from 0.75 to 0.94 is assumed, a Bayesian decision making model for waiting accurately reproduces behavioral data of DRN photostimulation. Blue and orange lines show the quitting time with and without increased prior probability, respectively. Blue- and yellow- shaded regions indicate distribution of waiting time during omission trials in activation and no-activation of DRN serotonergic neurons, respectively. (**B**) If an OFC photostimulation shift prior probability from 0.75 to 0.92 is assumed, the Bayesian decision making model correctly reproduces behavioral data of OFC photostimulation. Blue and orange lines show the quitting time with and without increased prior probability, respectively. Blue- and yellow-shaded regions indicate distribution of waiting time during omission trials in activation and no-activation of OFC serotonergic neurons, respectively. (**C**) Likewise, assuming an mPFC photostimulation shift prior probability from 0.75 to 0.85, the model successfully reproduces behavioral data of mPFC photostimulation in

the D4-6-8 and D2-6-10 tests, but not in the D6 and D10 tests. Blue and orange lines show the quitting time with and without increased prior probability, respectively. Blue- and yellow- shaded regions indicate distribution of waiting time during omission trials in activation and no-activation of mPFC serotonergic neurons, respectively. (**D**) Assuming an mPFC photostimulation shift prior probability from 0.75 to 0.80, the model also fails to fit behavioral data in the D4-6-8 and D2-6-10 tests. Blue and orange lines show the quitting time with and without increased prior probability, respectively. Blue- and yellow- shaded regions indicate distribution of waiting time during omission trials in no-activation and activation of mPFC serotonergic neurons, respectively. Red lines denote the internal model of reward-delivery timing, which is assumed to be a Gamma distribution with μ (6 in D6, D4-6-8, D2-6-10 tests and 10 in D10 test) and σ (1.5, 2.1, 3.3, and 2.7 in D6, D4-6-8, D2-6-10, and D10 tests, respectively).

Table S1. Mean waiting times and waiting-time ratios of the first model in which DRN, OFC, and mPFC photostimulation induce different prior reward probability shifts.

Simulation data					Behavioral data				
Operation	Test	Yellow	Blue	WTR	Operation	Test	Yellow	Blue	WTR
Prior probability shift 0.75 to 0.94	D6	10.61 s	11.97 s	1.13	DRN optognetic stimulation	D6	10.87 s	12.23 s	1.12
	D4-6-8	11.96 s	14.09 s	1.18		D4-6-8	11.87 s	14.14 s	1.19
	D2-6-10	14.62 s	18.67 s	1.28		D2-6-10	14.08 s	18.92 s	1.34
	D10	17.02 s	19.34 s	1.15		D10	17.55 s	19.62 s	1.12
Prior probability shift 0.75 to 0.92	D6	10.61 s	11.73 s	1.11	OFC optognetic stimulation	D6	10.79 s	12.03 s	1.12
	D4-6-8	11.96 s	13.72 s	1.15		D4-6-8	11.53 s	13.19 s	1.14
	D2-6-10	14.62 s	17.93 s	1.23		D2-6-10	13.77 s	16.68 s	1.21
	D10	17.02 s	19.12 s	1.12		D10	17.30 s	19.17 s	1.11
Prior probability shift 0.75 to 0.85	D6	10.61 s	11.16 s	1.05	mPFC optognetic stimulation	D6	11.06 s	11.22 s	1.01
	D4-6-8	11.96 s	12.82 s	1.07		D4-6-8	12.56 s	13.47 s	1.07
	D2-6-10	14.62 s	16.22 s	1.11		D2-6-10	14.72 s	16.43 s	1.12
	D10	17.02 s	18.05 s	1.06		D10	17.98 s	18.09 s	1.01
Prior probability shift 0.75 to 0.80	D6	10.61 s	10.86 s	1.02	mPFC optognetic stimulation	D6	11.06 s	11.22 s	1.01
	D4-6-8	11.96 s	12.36 s	1.03		D4-6-8	12.56 s	13.47 s	1.07
	D2-6-10	14.62 s	15.35 s	1.05		D2-6-10	14.72 s	16.43 s	1.12
	D10	17.02 s	17.50 s	1.03		D10	17.98 s	18.09 s	1.01

Yellow and Blue in simulation data indicate mean waiting time without and with prior reward probability shift, respectively. Yellow and Blue in behavioral data indicate mean waiting time without and with optogenetic stimulation, respectively. WTR: Waiting-time ratio. The standard deviations of the internal models of reward delivery timing were 1.5, 2.1, 3.3, and 2.7 sec in D6, D4-6-8, D2-6-10 and D10 tests, respectively.

Table S2. Mean waiting times and waiting-time ratios of the second model in which the OFC and mPFC calculate posterior reward probability separately.

Simulation data					Behavioral data				
Operation	Test	Yellow	Blue	WTR	Operation	Test	Yellow	Blue	WTR
Prior(OFC): 0.75 to 0.94 Prior(mPFC): 0.75 to 0.75	D6	10.44 s	11.77 s	1.13	OFC optognetic stimulation	D6	10.79 s	12.03 s	1.12
	D4-6-8	11.93 s	13.61 s	1.14		D4-6-8	11.53 s	13.19 s	1.14
	D2-6-10	14.39 s	17.35 s	1.21		D2-6-10	13.77 s	16.68 s	1.21
	D10	16.59 s	18.89 s	1.14		D10	17.30 s	19.17 s	1.11
Prior(OFC): 0.75 to 0.75 Prior(mPFC): 0.75 to 0.94	D6	10.44 s	10.56 s	1.01	mPFC optognetic stimulation	D6	11.06 s	11.22 s	1.01
	D4-6-8	11.93 s	12.89 s	1.08		D4-6-8	12.56 s	13.47 s	1.07
	D2-6-10	14.39 s	16.21 s	1.13		D2-6-10	14.72 s	16.43 s	1.12
	D10	16.59 s	16.98 s	1.02		D10	17.98 s	18.09 s	1.01
Prior(OFC): 0.75 to 0.94 Prior(mPFC): 0.75 to 0.94	D6	10.44 s	11.78 s	1.13	DRN optognetic stimulation	D6	10.87 s	12.23 s	1.12
	D4-6-8	11.93 s	14.08 s	1.18		D4-6-8	11.87 s	14.14 s	1.19
	D2-6-10	14.39 s	18.40 s	1.28		D2-6-10	14.08 s	18.92 s	1.34
	D10	16.59 s	18.95 s	1.14		D10	17.55 s	19.62 s	1.12

Yellow and Blue in simulation data indicate mean waiting time without and with prior reward probability shift, respectively. Yellow and Blue in behavioral data indicate mean waiting time without and with optogenetic stimulation, respectively. WTR: Waiting-time ratio. Prior(OFC): Prior probability of the OFC. Prior(mPFC): Prior probability of the mPFC. The standard deviations of the internal models of reward delivery timing for the OFC were 1.5, 2.0, 3.0, and 2.6 sec in D6, D4-6-8, D2-6-10 and D10 tests, respectively. The standard deviations of the internal models of reward delivery timing for the mPFC were 1.0, 2.4, 4.0, and 2.0 sec in D6, D4-6-8, D2-6-10 and D10 tests, respectively.
